# Incorporating respiratory signals for machine learning-based multimodal sleep stage classification: a large-scale benchmark study with actigraphy and heart rate variability

**DOI:** 10.1093/sleep/zsaf091

**Published:** 2025-04-11

**Authors:** Daniel Krauss, Robert Richer, Arne Küderle, Jelena Jukic, Alexander German, Heike Leutheuser, Martin Regensburger, Jürgen Winkler, Bjoern M Eskofier

**Affiliations:** Machine Learning and Data Analytics Lab, Friedrich-Alexander-Universität (FAU) Erlangen-Nürnberg, Erlangen, Germany; Machine Learning and Data Analytics Lab, Friedrich-Alexander-Universität (FAU) Erlangen-Nürnberg, Erlangen, Germany; Machine Learning and Data Analytics Lab, Friedrich-Alexander-Universität (FAU) Erlangen-Nürnberg, Erlangen, Germany; Department of Molecular Neurology, Universitätsklinikum Erlangen, Elangen, Germany; Department of Molecular Neurology, Universitätsklinikum Erlangen, Elangen, Germany; Machine Learning and Data Analytics Lab, Friedrich-Alexander-Universität (FAU) Erlangen-Nürnberg, Erlangen, Germany; Department of Molecular Neurology, Universitätsklinikum Erlangen, Elangen, Germany; Department of Molecular Neurology, Universitätsklinikum Erlangen, Elangen, Germany; Machine Learning and Data Analytics Lab, Friedrich-Alexander-Universität (FAU) Erlangen-Nürnberg, Erlangen, Germany

**Keywords:** machine learning, deep learning, wearable electronic devices, multimodal sensing, neural networks, sleep, sleep-stage

## Abstract

Insufficient sleep quality is directly linked to various diseases, making reliable sleep monitoring crucial for prevention, diagnosis, and treatment. As sleep laboratories are cost- and resource-prohibitive, wearable sensors offer a promising alternative for long-term unobtrusive sleep monitoring at home. Current unobtrusive sleep detection systems are mostly based on actigraphy (ACT) that tend to overestimate sleep due to a lack of movement in short periods of wakefulness. Previous research established sleep stage classification by combining ACT with cardiac information but has not investigated the incorporation of respiration in large-scale studies. For that reason, this work aims to systematically compare ACT-based sleep-stage classification with multimodal approaches combining ACT, heart rate variability (HRV) as well as respiration rate variability (RRV) using state-of-the-art machine- and deep learning algorithms. The evaluation is performed on a publicly available sleep dataset including more than 1000 recordings. Respiratory information is introduced through ECG-derived respiration features, which are evaluated against traditional respiration belt data. Results show that including RRV features improves the Matthews Correlation Coefficient (MCC), with long short-term memory (LSTM) algorithms performing best. For sleep staging based on AASM standards, the LSTM achieved a median MCC of 0.51 (0.16 IQR). Respiratory information enhanced classification performance, particularly in detecting wake and rapid eye movement (REM) sleep epochs. Our findings underscore the potential of including respiratory information in sleep analysis to improve sleep detection algorithms and, thus, help to transfer sleep laboratories into a home monitoring environment. The code used in this work can be found online at https://github.com/mad-lab-fau/sleep_analysis.

## Graphical Abstract

**Graphical Abstract:**
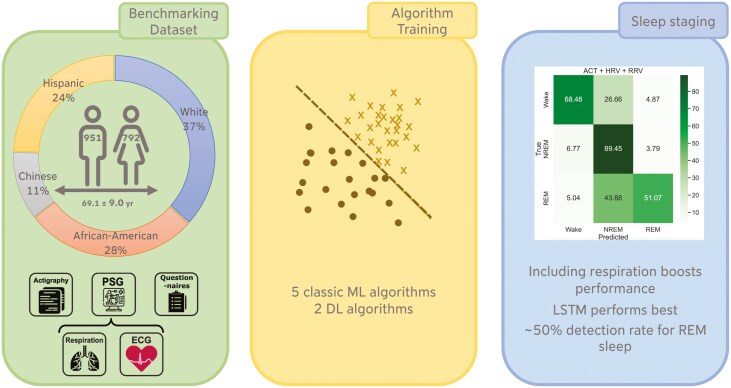

**Statement of Significance**

We present a systematic comparison of different ML and DL algorithms for automated sleep stage classification from different sensor modalities. We evaluated the performance of these algorithms based on ACT- and HRV-based features and investigated whether adding respiratory information can enhance classification performance. By incorporating respiratory information derived from a respiration belt or ECG signals, our work demonstrates significant improvements in sleep stage detection, especially for rapid eye movement sleep and wakefulness. We provide a fully reproducible open-source implementation of our analysis as well as a reusable framework for preprocessing raw sensor data and extracting features from ACT, ECG, and respiration signal, as well as a framework for calculating EDR from raw ECG and then calculating ED-RRV features from that.

Sleep affects a variety of daily activities such as learning, attention, productivity, and memory [1]. Insufficient sleep is directly linked to a series of diseases like diabetes or hypertension and leads to a higher risk of strokes [1, 2]. Furthermore, specific sleep patterns, such as rapid eye movement (REM) behavior disorder (RBD), can be an indicator for neurodegenerative diseases such as Parkinson’s disease [3].

For that reason, sleep monitoring is crucial for many application scenarios in medicine and psychology as it can help to identify the causes of sleep disorders, thus enabling to initiation of adequate therapies.

The gold standard approach for sleep monitoring and the detection of sleep disorders is polysomnography (PSG), which is typically performed in a sleep laboratory [4]. In a PSG examination, different physiological signals like electroencephalogram (EEG), electrocardiogram (ECG), and respiration are assessed during sleep, as well as body orientation and muscle activities of limbs [4]. These signals are then used to assess certain characteristics during sleep such as obstructive sleep apnea or the distribution of sleep phases. Usually, the sleep stage labels from PSG recordings are divided into epochs of 30 seconds or 1 minute. These epochs are then labeled as sleep phases by a trained professional. Thereby, the EEG signals, which provide information about the electrical activity of the brain, serve as the primary basis for determining sleep stages.

According to the American Academy of Sleep Medicine (AASM), sleep can be divided into two fundamental types: REM, which is associated with active dreaming, and non-rapid eye movement (NREM) which can be further divided into the three stages N1–N3.

While the high precision of PSG allows a good and reliable diagnosis of sleep disorders, it also suffers from several drawbacks. Primarily, longitudinal measurements are impractical since PSG is very cost- and resource-intensive. Furthermore, the unfamiliar laboratory setting can influence the sleep quality of patients causing unrealistic sleep patterns [5].

In contrast, sleep diaries are inexpensive and easy to acquire for large-scale datasets. However, sleep diaries can, at best, only measure the distribution of sleep and wake phases, but cannot further examine the distribution of the different sleep stages. Furthermore, sleep diaries lack accuracy because of imprecise data during the night, poor awareness of exact sleep and wake times, as well as a subject-specific bias [6, 7].

To improve patient experience and prevent diseases at an early stage, wearable sensors, such as motion or ECG sensors offer a promising alternative [8]. Since these sensor systems are broadly accepted in the population, unobtrusive, and low-cost, individuals can follow their regular daily habits and, most importantly, sleep in their own beds, facilitating sleep monitoring in a more realistic setting. These advantages enable longitudinal studies, thus potentially allowing to development of better diagnostic approaches. However, wearable sensor systems usually don’t include an EEG, as placing sensors at the head is obtrusive and labor-intensive. Hence, these systems need to rely on secondary physiological processes to detect sleep stages characterized by brain wave activity.

During sleep, body movements decrease compared to a wakeful state [9]. Therefore, various algorithms were developed that examine sleep analysis based on actigraphy (ACT). ACT refers to the aggregated physical movement, typically captured from wrist-worn smartwatches, which is sampled multiple times per second and aggregated in epochs of 30 seconds or 1 minute. Using this information, sleep–wake detection can be performed achieving accuracies greater than 80% [10–14].

However, concurrently with falling asleep, not only the amount of movement but also cardiac and respiratory activity changes. Throughout the different sleep stages, various body functions are reduced [1, 15, 16]. Within NREM-phases, blood pressure decreases by 10% concurrently with a lower heart rate, heart rate variability (HRV), and a 73% decreased tidal volume, the amount of air inhaled and exhaled during each breath [17–20]. During wakefulness, breathing tends to be irregular, while in NREM sleep, it becomes more regular as the sleep deepens. Conversely, during REM sleep, breathing patterns become more irregular with short breaks in breathing. Additionally, the tidal volume is smaller during REM sleep as compared to NREM sleep [19, 21].

To classify sleep phases based on physiological signals other than EEG, different types of algorithms can be used. Traditionally, unobtrusive sleep detection using wearable sensors was solely based on ACT using heuristic algorithms. Initially, these algorithms were rule-based and examined only the detection of sleep and wake phases. Prominent heuristic algorithms have been developed by Webster et al. [10], Kripke et al. [11], and Cole et al. [12]. These threshold-based algorithms work with ACT data that are convolved with a windowed kernel to gain time dependency. However, these heuristic algorithms are not trainable, and adapting to complex sleep patterns is difficult. Furthermore, due to decreased body movement in short periods of wakefulness, ACT-only-based algorithms tend to overestimate sleep [22]. To get a better insight into different sleep phases, data-driven approaches using different Machine Learning (ML), and Deep Learning (DL) algorithms were developed, outperforming the commonly used heuristic ones.

Imtiaz et al. [23] reviewed 90 different papers about wearable sensing technologies for sleep-stage classification and identified 13 different input modalities used in clinical and home monitoring environments. The most prominent input modalities for unobtrusive sleep analysis are motion and cardiac data. Perez-Pozuelo et al. [7] used wearable ECG and photo-plethysmography (PPG) sensors to detect sleep and wake phases and compared the results to sleep diaries and PSG annotations. They reported higher agreement rates in detecting sleep windows compared to sleep diaries. However, this work did not classify wake phases during the night and did not distinguish different sleep stages. Radha et al. [24] provided a sleep-stage classification system based on HRV using long short-term memory (LSTM) networks. Even though they found promising results, their classification performance declined for participants aged above 50 who represent the primary target demographic for sleep monitoring as sleep diseases are most prevalent in this age group [25]. Zhang et al. [26] developed an end-to-end learning framework to extract sleep stages from heart rate data and wrist actigraphy and reported promising results without expert feature engineering.

Another technique to unobtrusively obtain information about sleep is contactless measurement using radar signals [27]. Recent research reported promising results in sleep stage classification based on movement, cardiac, and respiratory information extracted from different radar sensing techniques [28–30].

In order to determine the best set of algorithms for sleep classification, it is important to systematically compare different algorithms with different input modalities on a large-scale, diverse, and publicly available dataset. This ensures comparability between different approaches and avoids the risk of study-specific influences. Palotti et al. [31] compared various state-of-the-art heuristic, ML, and DL algorithms for sleep–wake detection on a large-scale dataset while Zhai et al. [32] assessed different state-of-the-art ML- and DL algorithms to classify sleep stages using HRV and ACT in mono- and multimodal approaches. They found that ML and DL algorithms performed considerably better than heuristic algorithms, while the addition of cardiac information improved sleep stage classification.

As respiratory characteristics change within the different sleep phases, incorporating respiratory information into the classification pipeline might enhance the classification performance. Previous work [33, 34] shows promising results in sleep–wake and sleep-stage classification based on multimodal approaches, combining respiratory features and ACT. However, those experiments were only trained and tested on a small number of participants. Furthermore, Sun et al. [35] performed sleep staging based on ECG recordings, abdominal and chest respiration from a respiration belt alone, as well as with the combination of ECG-recordings and the respiratory data. They found improvements by including both sensor modalities instead of just ECG or respiration but similar results for abdominal and chest respiration. Gaiduk et al. [36] performed sleep staging based on movement, heart rate, and respiratory signals and confirmed the potential of unobtrusive sleep monitoring using these modalities. However, this study was only performed within a small cohort and did not investigate how the single modalities influence the classification results.

Extracting respiratory information from an additional sensor, such as a respiration belt or a nasal cannula, increases obtrusiveness and might lead to negative influences on sleep quality and user acceptance. To obtain respiratory information without introducing additional sensors, inhalation and exhalation can be derived from other sensing modalities, such as from accelerometers or from ECG via ECG-derived respiration (EDR) [37–39].

Due to inhalation and exhalation, the ECG signal gets modulated. Within the breathing cycle, the baseline of the signal changes due to changes in electrode-skin impedance and slight shifts in electrode positioning (baseline wander). During inspiration, the lungs expand, increasing the volume of air in the thoracic cavity leading to increased impedance. This can lead to a decreased amplitude of the QRS complex and the T-wave (amplitude modulation). Another modulation named respiratory sinus arrhythmia is caused by changes in heart rate due to the ANS. During inhalation, the activity of the parasympathetic nervous system decreases, leading to a temporary increase in heart rate. During exhalation, the parasympathetic activity increases, which slows down the heart rate (frequency modulation) [40].

While prior research used ACT, HRV, and respiratory information to classify sleep stages [26, 32, 33, 36, 39], large benchmark studies often compare a limited number of modalities or algorithms without exploring the synergies between different biosignals. For instance, it has not yet been investigated whether integrating respiratory information with HRV and movement data can enhance sleep stage classification beyond the established combinations of actigraphy (ATC) and HRV alone. To address this gap, this work systematically compares different ML and DL algorithms for sleep stage detection using different sensor modalities on the MESA Sleep dataset [41, 42]—one of the largest open-access datasets that includes both PSG and ACT data. We evaluated these algorithms in ablation studies using ACT data established ACT-HRV-based methods, and assessed the impact of incorporating respiratory information. Additionally, to reduce the number of sensors and participant burden, we substituted respiration belt data with respiratory information extracted via EDR and systematically compared the results with the approaches using respiratory information extracted from a respiration belt.

## Methods

### Dataset

The Multi-Ethnic Study of Atherosclerosis (MESA) dataset is one of the largest open-access datasets that combines gold standard measurements of PSG with ACT [41, 42]. The MESA study is a longitudinal investigation of factors associated with sub-clinical and clinical cardiovascular disease. However, the study did not specifically enrich individuals with atherosclerosis [43]. Compared to the original cohort, there was a minor enrichment of younger mean age (68 years in participants vs. 71 years in non-participants), black ethnicity (28.0% vs. 25.5%), nonsmokers (93.5% vs. 92.3%) and persons without arterial hypertension (43.3% vs. 38.0%). The study, conducted at six centers in the United States included 6814 men and women of different ethnicities. Of these, 2237 participants were enrolled in a sleep exam (MESA Sleep) which included a sleep questionnaire, unattended full overnight PSG recordings, ACT recordings, ECG data, and respiratory information acquired from a thoracic respiratory belt via respiratory induction plethysmography.

The ACT was collected using wrist-worn devices (Actiwatch Spectrum, Philips Respironics, Cambridge, USA) that record movements and aggregate them to activity counts in 30-second epochs. To record an in-home PSG, the Compumedics Somte System (Compumedics Ltd., Abbotsford, Australia) was used. The PSG incorporated cortical EEG, bilateral electrooculography, chin electromyography, thoracic and abdominal respiratory inductance plethysmography, airflow, ECG, leg movements, and finger pulse oximetry. Details of sampling rates, scoring rules, and data collection protocols are available in the dataset description ^1^. Nocturnal recordings were transmitted to the centralized reading center at Brigham and Women’s Hospital (Boston, USA) and the sleep stages were scored by trained technicians, based on EEG and EOG, following current guidelines. The QRS-complexes and R-peaks, which were already extracted within the dataset, were detected from the ECG signal using the Compumedics (Abbotsford, VIC, Australia) Somte software Version 2.10 (Builds 99 to 101) and reviewed by a trained technician who corrected misscored annotations during the sleep period [41, 42].

We excluded 497 of the 2237 participants in the dataset because (1) no PSG, ECG, or ACT recordings were available, (2) no overlap of PSG and ACT was specified, (3) no respiration signal was extractable from the PSG recordings, or (4) the total sleep time was less that 2 hours. Additionally, 620 participants were excluded due to low-quality PSG or ACT recordings specified in the metadata, resulting in a final sample size of 1,120 participants with high-quality overnight sleep recordings.

**Figure 1.**
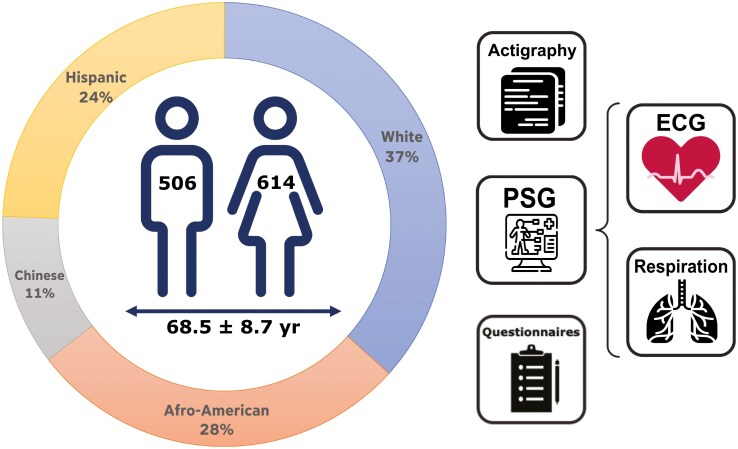
Illustration of the datastreams used from the MESA sleep dataset.

We randomly assigned 80% of the participants (*N* = 896) to a training set and 20% (*N* = 224) participants to a test set for the ML algorithms. Further details regarding the age, gender, ethnicity, and sleep statistics of the participants in the train- and test dataset are provided in Figure 1 and Table 1, respectively.

**Table 1:** Statistics of the MESA Sleep Dataset, age (𝑈 = 9.97 * 10^4^, 𝑝 = .884, 𝑔 = 0.497), Total Sleep Duration (TSD; 𝑈 = 9.60 * 10^4^, 𝑝 = .311, 𝑔 = 0.4781), Wake After Sleep Onset (WASO; 𝑈 = 9.45 * 10^4^, 𝑝 = .174, 𝑔 = 0.471), and Sleep Efficiency (SE; 𝑈 = 1.07 * 10^5^, 𝑝 = 0.145, 𝑔 = 0.532) Are Given As Mean ± SD

Dataset	Total	Male/Female	Age	TSD (min)	WASO (min)	SE [%]
**Train**	896	413 (46.1%)/483 (53.9%)	68.5 ± 8.8	485.1 ± 80.3	73.8 ± 53.1	76.9 ± 12.1
**Test**	224	93 (41.5%)/131 (58.5%)	68.5 ± 8.4	491.6 ± 76.5	80.8 ± 59.4	75.5 ± 13.0

### Preprocessing

We cleaned and filtered the RR-Intervals, which were already extracted and available in the dataset, by removing outliers, interpolating missing values, and removing ectopic beats using the Python package hrvanalyis ^2^. Afterward, the NN-intervals (i.e. RR-intervals of “normal” beats) were grouped into 30-second epochs to match data obtained by PSG and ACT. To ensure that only overlapping epochs were considered, ACT, PSG, and NN-intervals were aligned using metadata information from the MESA Sleep dataset. Moreover, we cut the PSG recording to the time window the participants were in bed, according to the metadata.

To obtain respiratory information we extracted respiratory induction plethysmography data from the PSG recordings. We applied a second-order bandpass Butterworth filter (0.1–0.35 Hz) to clean the respiratory signal, followed by the removal of the baseline drift implemented in the python package biosppy [44]. We then downsampled the data from 256 Hz to 32 Hz to reduce computational complexity and normalized the signal to a range between 0 and 1.

### EDR extraction

**Figure 2.**
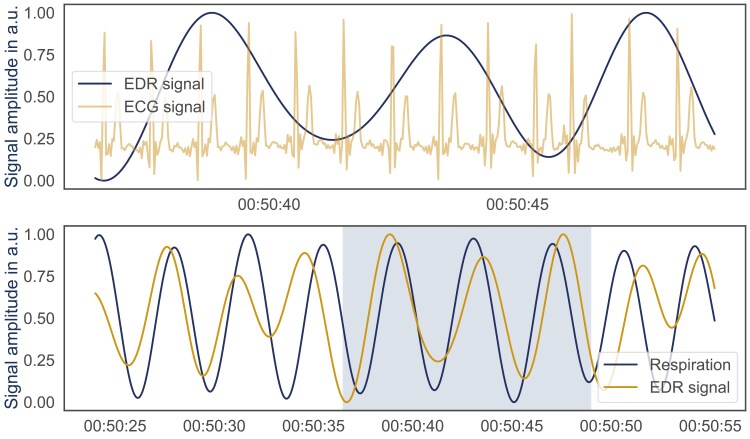
Comparison of respiration waveforms and ECG signals. The upper plot shows the respiration signal alongside the concurrently recorded one-lead ECG signal. The lower plot compares the respiration signal with the EDR signal extracted from ECG. The shaded region in the lower plot corresponds to the time segment depicted in the upper plot.


In addition to using the gold standard respiration signal extracted from respiratory induction plethysmography, we additionally estimated respiratory information from the ECG using EDR (Figure 2). For this purpose, we cleaned and filtered the raw ECG signal using the python package biopsykit [45]. To estimate the respiration signal we used an approach based on the amplitude of the QRS complex initially proposed by Charlton et al. [46]. Therefore, we calculated the mean amplitude between R-peaks and minima before R-peaks (troughs). Afterward, we normalized the resulting EDR signal to a range between 0 and 1:

$$\begin{array}{l} r_{\mu }=\;\frac{r_{raw}-r_{min}}{r_{max}-r_{min}} \end{array}$$

After normalization, we cleaned and downsampled the EDR signal in the same way as explained above for the respiration signal collected via the respiration belt.

### Feature extraction

To assess the potential to analyze sleep using information about movement, cardiac activity, and respiration, we trained the ML models in different multimodal approaches. Therefore, we extracted domain-specific expert features for ACT, ECG, and respiration data. As sleep stages are not instantaneous but evolve over time, we capture the temporal context by applying window-wise feature extraction [47].

We extracted a total of 370 time-series movement features based on ACT data. These features were computed using centered and non-centered sliding windows of varying sizes ranging from 30 seconds to 10 minutes (Table S1).

Additionally, we extracted 30 HRV features that describe the variability of beat-to-beat intervals, calculated from the time interval between several R-peaks. Similar ACT and HRV features were also commonly used in previous works [24, 31, 32, 48] (Table S2).

To characterize respiratory activity in the single sleep phases, we extracted respiration rate variability (RRV) features that contain information about variations in the rate and rhythm of breathing over time. We extracted these RRV features for centered sliding windows of 5, 7, and 9 minutes with an overlap of *window size—30 seconds* according to Fonseca et al [49]. Within these sliding windows, we extracted the peaks and troughs and computed 60 different RRV features using the Neurokit2 library [50, 51], which are listed in Table S3. Due to the overlap, the features were grouped into epochs of 30 seconds to match the datastreams from the movement and HRV features, as well as the expert-labeled sleep phases from PSG. The ED-RRV features were extracted from the EDR signal using the same approach. Feature rows for sliding windows where no respiration could be obtained due to noisy ECG data were filled with zeros.

For the DL algorithms, we did not perform any high-level feature extraction since neural networks have the ability to implicitly learn abstract concepts [52]. Thus, raw activity counts were used as input for the movement-based approach, whereas eight basic HRV features served as cardiac input for the DL algorithms according to Zhai et al. [32]. To also include respiratory information, four relatively simple RRV or ED-RRV features were used as input (Table 2).

**Table 2.** Modalities and Input Data for Deep Learning Models

Modality	Input data
ACT	Raw activity counts
HRV	Time-domain: Mean NN, SDNN, SDSD Frequency-domain: VLF, HF, LF, LF/HF ratio, total power
RRV/ED-RRV	Time domain: MeanBB_5min Frequency-domain: LF_5min, HF_5min, LFHF_5min

The full feature names can be found

in Tables S1, S2, and S3.

### Model- and hyperparameter training

**Figure 3.**
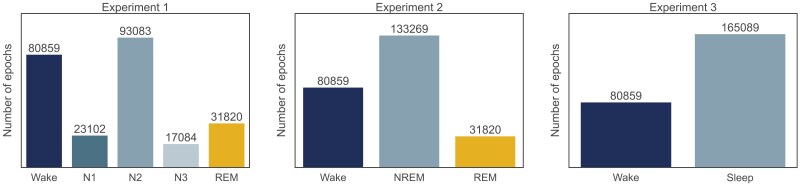
The distribution of wake and sleep epochs in the Test set is dependent on classification granularity.

To evaluate the performance of different ML and DL algorithms for classifying sleep stages, we trained various models using different granularities of sleep stages and modalities. Specifically, we trained five ML algorithms, including (1) Support Vector Machine (SVM), (2) Multilayer Perceptron (MLP), (3) Adaptive Boosting (AdaBoost), (4) Random Forest, and (5) XGBoost, as well as two DL algorithms, including (1) long short-term memory and (2) Temporal Convolutional Network (TCN). By including these different algorithms, we ensure to cover a diverse set of frequently used algorithmic families: linear methods that are known as robust baseline classifier (SVM), fully connected neural networks (MLP), ensemble classifiers (AdaBoost, Random Forest, XGBoost), and DL methods specifically suitable for modeling time series (LSTM, TCN). Those models were also frequently used in different previous studies [28, 31, 32] making our findings more comparable to literature. Figures S1 and S2 in the Appendix display a visualization of the LSTM and TCN model architectures. We trained these algorithms on three different granularities of sleep stages, including (1) wake/REM/N1/N2/N3, (2) wake/REM/NREM, where NREM includes N1, N2 and N3 epochs, and (3) sleep–wake detection. Figure 3 displays the distribution of wake and sleep epochs dependent on the granularity of sleep stages.

Furthermore, we employed all algorithms with different combinations of input modalities, including (1) ACT-only, (2) ACT + HRV, (3) ACT + HRV + RRV, and (4) ACT + HRV + ED-RRV. We conducted a systematic comparison of the classification results obtained from each algorithm and modality combination to identify the most promising approach for sleep stage classification.

In order to find the best set of hyperparameters for AdaBoost, MLP, and SVM, we performed a grid-search with an embedded five-fold cross-validation (CV) within a predefined parameter space. As SVM and MLP models rely on distance calculations between data points, we standardized the features using z-score standardization of the training data within each CV fold. Furthermore, we performed select k-best feature selection for those algorithms and treated k as one of the hyperparameters in the optimization process. Due to the large number of hyperparameters for Random Forest and XGBoost and the associated computational cost, we employed a 250 (Random Forest) and 50 (XGBoost) trial hyperparameter optimization with an embedded five-fold CV using the optuna library [53]. Specifically, we applied a Tree-structured Parzen Estimator to select hyperparameters from a predefined search space based on previous trials [54]. These optimizations were performed within the tpcp framework [55].

Due to the computational cost of DL algorithms, we performed no CV. Instead, we optimized the model using a single validation set separated from the training set via an 80/20 split, resulting in a total of 717 participants used for training and 179 for validation. For a defined number of epochs, we applied the validation set on the current model and calculated the corresponding loss. Each time a new minimum validation loss was found, we saved the model and finally evaluated the best model on the test set. To find the optimal hyperparameters for the DL algorithms, we also employed a Tree-structured Parzen Estimator within the optuna framework [53, 54]. The hyperparameters which were optimized are listed in Table S4 and Table S5.

For the DL-based algorithms, we first applied a centered windowing approach to the data, using different sequence lengths of 10, 25, and 50 minutes, looking at half the window size backward and forward from the center. Each sequence was treated as a hyperparameter and extracted on a participant-wise basis to ensure that data within each window came from a single participant only. To standardize the input data across all windows, we applied z-normalization. For the epoch-wise training of the DL networks, we used adaptive moment estimation (Adam), a gradient descent-based optimization method that computes adaptive learning rates for each parameter. Additionally, we performed a 250-trial hyperparameter search using a Tree-structured Parzen Estimator and the optuna library [53, 54] to identify the optimal combination of hyperparameters, such as the learning rate, number of layers, and hidden layer size.

### Code availability

In order to ensure the transparency and reproducibility of our results, we made the code used in this study available on GitHub: https://github.com/mad-lab-fau/sleep_analysis. This software is implemented in Python with additional Jupyter notebooks used for the evaluation. Our code includes the preprocessing steps of raw sensor data, feature extraction from ACT, ECG, and respiration, as well as the implementation of the ML and DL algorithms used in this study. We also provide a framework for calculating EDR from raw ECG and calculating ED-RRV features. In addition, we included the implementation of five commonly used heuristic algorithms for sleep–wake classification based on ACT, which were not considered in this study. We conducted all experiments on data from the MESA-Sleep dataset, which is publicly available on sleepdata.org allowing others to replicate our study and build upon our findings. Furthermore, other researchers can build upon our findings and include additional biosignals, such as PPG-based features. Furthermore, this code can be used to train baseline models on a large-scale dataset and finetune them on other smaller datasets. Notably, the dataset follows the structure of the Python package tpcp ^3^ making it easy for researchers to test the framework with their own data or other benchmark datasets.

## Evaluation

The test dataset was exclusively used to evaluate the performance of the trained models. Since sleep data collected during the night are usually unbalanced, optimizing for accuracy is not feasible. For that reason, the performance of the ML and DL algorithms were optimized with regard to the Matthews Correlation Coefficient (MCC), which is known to be more meaningful to imbalanced datasets compared to classical measures such as accuracy, as it accounts for all elements of the confusion matrix [56]. However, it does not directly mitigate class imbalance itself. Equation 1 presents the formula for calculating MCC where TP, FP, TN, and FN represent *true positives, false positives, true negatives*, and *false negatives*, respectively.


$$\begin{array}{l} MCC\;=\;\frac{TP*TN-FP*FN}{\sqrt{\left(TP+FP)(TP+FN)(TN+FP)(TN+FN\right)}} \end{array}$$ (1)

Although accuracy is not the primary optimization metric, it is still important for assessing the overall proportion of correctly classified epochs and for comparison with previous studies. Therefore, we calculated and reported accuracy in addition to other performance metrics, including the F1-score, precision, recall, and specificity, which are all derived from the confusion matrix.

We assessed sleep stage detection across three configurations, referred to as experiments 1, 2, and 3 in the results section. Experiment 1 involved classifying according to AASM standards into five distinct sleep stages: wake, N1, N2, N3, and REM. Experiment 2 simplified this to a three-class classification of wake, REM, and NREM stages, whereas NREM was defined as the combination of N1, N2, and N3 epochs. Experiment 3 further reduced this to a binary classification between sleep and wake stages. For the ML and DL algorithms, we determined the best set of hyperparameters by the combination which achieved the highest average MCC over the five folds for the ML algorithms and the highest MCC in the validation set for the DL algorithms. We then retrained the classifier with this hyperparameter set on the complete training data and applied this retrained model to the test set.

To test differences found between different algorithms and modalities, we applied the Mann–Whitney-*U* Tests [57] with Bonferroni correction for multiple-comparison correction. We set the significance level to *alpha = .05*. In all Figures and Tables, we used the following notation to indicate statistical significance: *p < .05, **p < .01, ***p < .001.

## Results

The main objective of this work was to investigate whether respiratory information can help to improve classification performance compared to the already established combination of ACT and HRV. For that purpose, we compared the performance of seven state-of-the-art ML and DL algorithms for sleep stage classification using the different combinations of input modalities and different granularities of sleep stages. Detailed results of all experiments can be found in Tables S6–S8. Each Table corresponds to a different granularity of sleep stages. For each evaluation metric, the best-performing algorithm is written in bold. The best-performing ML and DL algorithms with respect to MCC are presented as boxplots.

### Experiment 1: wake/N1/N2/N3/REM classification (AASM standard)

Our results show that the LSTM-based algorithm outperformed all other algorithms in terms of MCC classification performance across all tested combinations of input modalities. The XGBoost algorithm demonstrated the best performance among the ML algorithms. However, all ML algorithms performed within the same range with respect to MCC.

The addition of HRV features to ACT significantly (*p < .001*) influenced the classification performance in DL algorithms with higher median values observed (+12.9 percentage points F1-score). In contrast, the improvement in XGBoost performance with this combination was relatively minor (+3.1 percentage points F1-score).

By further incorporating RRV features to ACT and HRV features, we found significant changes for XGBoost (*p < .001*) with strong improvement in median performance (+8.2 percentage points F1-score). In contrast, the DL algorithms only showed small improvements (LSTM: + 0.7 percentage points F1-score).

**Figure 4.**
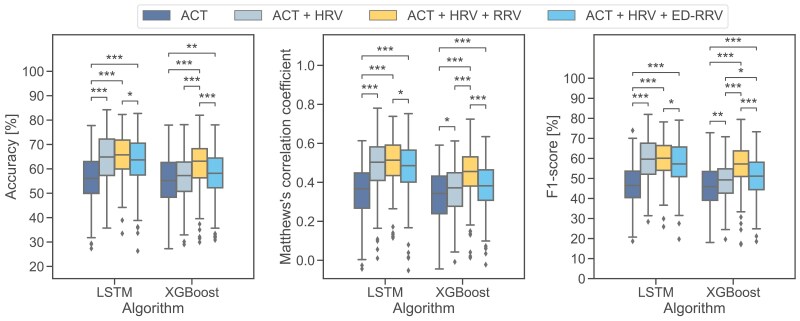
Performance of sleep-stage classification according to AASM standards (wake/N1/N2/N3/REM) of the best-performing ML (XGBoost) and DL (LSTM) algorithms. *p < .05, **p < .01, ***p < .001.

Including respiratory information via ED-RRV features instead of respiratory information from a respiration belt yielded slightly lower results compared to the ACT + HRV approach for DL algorithms, but a moderate increase in performance for ML algorithms (Figure 4). Detailed results can be found in Table S6.

**Figure 5.**
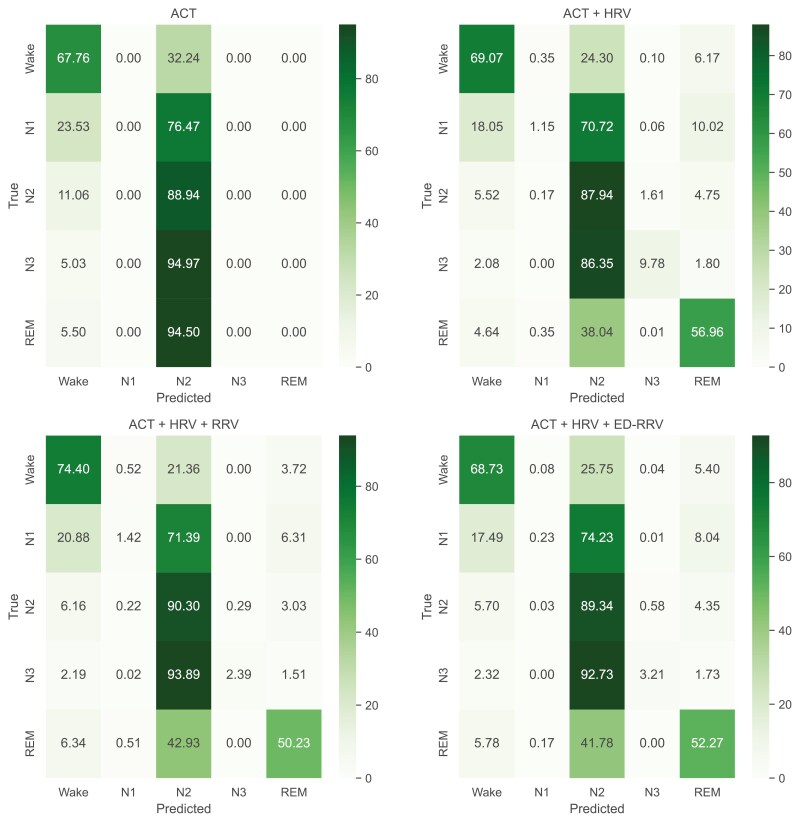
Confusion-matrix of sleep staging via LSTM classification according to AASM standards (wake/N1/N2/N3/REM). Values are given in %.

Figure 5 displays the confusion matrix of the best-performing DL algorithm (LSTM). We found that using ACT-only for classification cannot distinguish between N1, N2, N3, and REM sleep epochs. However, adding HRV features into the classification pipeline enhanced the detection of REM sleep, achieving 56 % correctly classified epochs. Further adding RRV features led to an improvement in wake classification (+5.3 percentage points). However, the classification of N3 sleep phases decreased (−7.4 percentage points).

**Figure 6.**
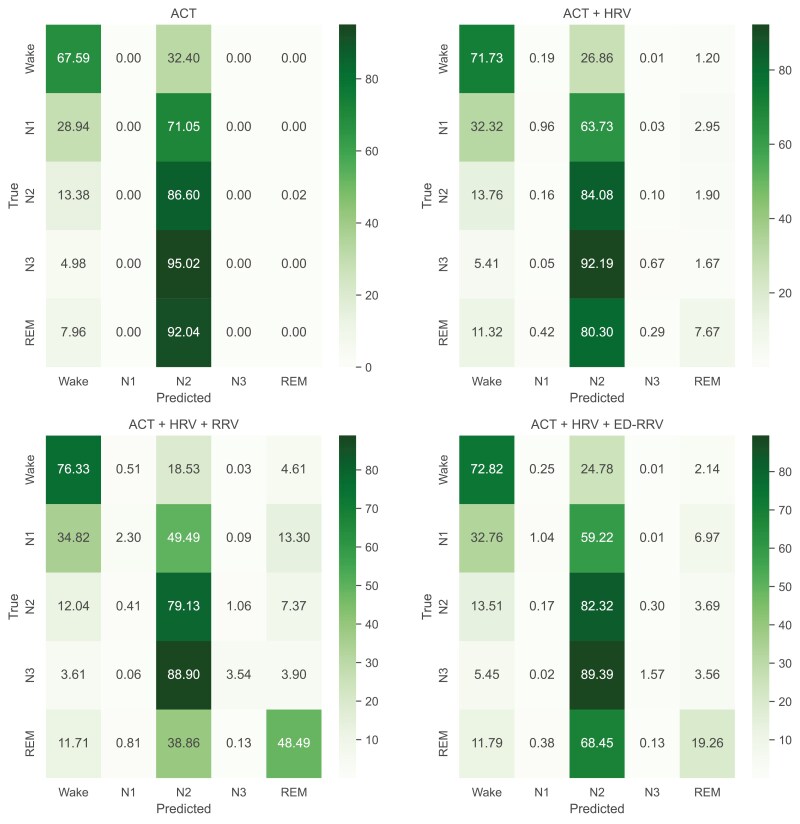
Confusion-matrix of sleep staging via XGBoost classification according to AASM standards (wake/N1/N2/N3/REM). Values are given in %.

The confusion matrix for classification using XGBoost is depicted in Figure 6. Including HRV features improved wake classification (+4.1 percentage points), but did not substantially improve the classification of N1, N3, and REM epochs. However, by adding RRV features to the ACT + HRV approach we noticed remarkably more correctly classified epochs of REM sleep (+40.5 percentage points) and a better detection of epochs when the individual was awake (+4.6 percentage points).

Interestingly, the incorporation of ED-RRV features led to comparable classification performances in Wake and NREM epochs, while the detection of REM sleep shows substantial improvements (+11.6 percentage points).

### Experiment 2: wake/non-REM/REM classification

**Figure 7.**
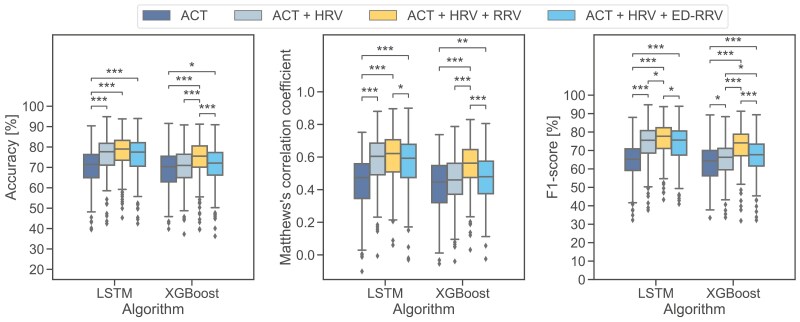
Performance of wake/NREM/REM classification of best ML (XGBoost) and DL (LSTM) algorithms. *p < .05, **p < .01, ***p < .001.

Consistent with the results of the first experiment, the LSTM-based algorithm performed best across all input modalities (Figure 7 and Table S7).

By introducing HRV to the ACT features, the DL-based algorithms showed significantly better results (*p < .001*) with an increase in the median F1-score (LSTM: + 10.2 percentage points). In contrast, the ML algorithms only improved slightly (XGBoost: + 2.0 percentage points in F1-score). Adding respiratory information via RRV features led to small improvements in the DL algorithms (LSTM: + 2.2 percentage points F1-score) but significant differences (*p* < .001) with a higher median F1-score for ML algorithms (XGBoost: + 7.8 percentage points).

By introducing respiratory information via ED-RRV instead of RRV features we observed a moderate increase in classification performance for the ML algorithms. As already found in the first experiment, this improvement was primarily due to a better detection of REM sleep epochs. However, for the DL algorithms, adding ED-RRV features to ACT and HRV features even slightly decreased the classification performance.

Detailed confusion matrices can be found in Figures S3 and S4.

### Experiment 3: sleep–wake classification

**Figure 8.**
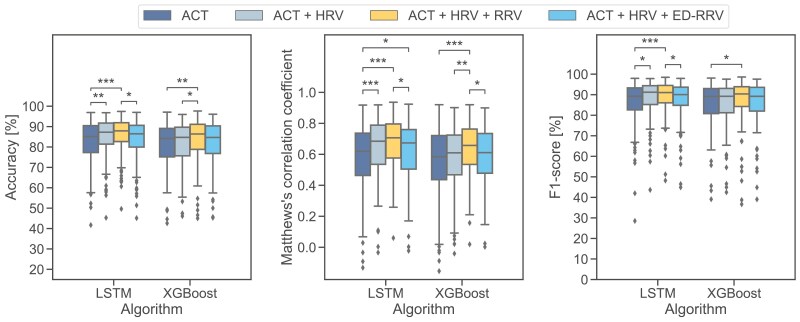
Performance of sleep–wake detection of best ML and DL algorithm. *p < .05, **p < .01, ***p < .001.

Table S8 summarizes the results of our binary classification approach, while Figure 8 shows the performance of the best ML and DL classifiers.

The LSTM achieved the highest performance measures except for recall. In particular, the model showed high performance in specificity compared to the other algorithms, which can be used to measure the ability to avoid overpredicting sleep stages in the binary classification task. Among the ML algorithms, the XGBoost classifier performed best, although all ML algorithms showed similar performance levels. As already demonstrated by previous research [31, 32], several key performance metrics for sleep–wake detection of the best ML (XGBoost) and DL (LSTM) algorithm significantly (*p < .05*) improved by adding HRV features to the ACT-only approach.

Further including RRV features into the classification model led to another increase in median F1-score in classification performance for XGBoost (+1.2 percentage points) but comparable performance for the LSTM (−0.2 percentage points F1-score). The incorporation of ED-RRV features instead of RRV led to comparable results for LSTM (−0.1 percentage points F1-score) and XGBoost (−0.6 percentage points F1-score; Table S8). Detailed confusion matrices can be found in Figures S5 and S6.

### Influences on classification performance

To identify possible influences on classification performance, we conducted several statistical tests to correlate classification performance with available metadata in the dataset.

**Figure 9.**
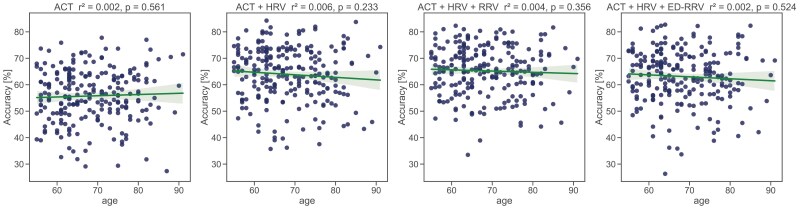
Linear regression of age and classification accuracy of the best-performing model (LSTM) in the wake/N1/N2/N3/REM classification approach.

**Figure 10.**
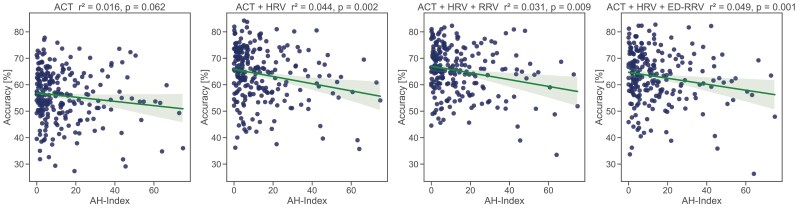
Linear regression of apnoea–hypopnoe index and classification accuracy of the best-performing model (LSTM) in the wake/N1/N2/N3/REM classification approach.

We found that the performance is robust across different age groups (Figure 9), but decreases for patients diagnosed with a high apnoe–hypopnoe index (Figure 10).

We also examined the effects of gender, origin, and the diagnosis of insomnia and restless legs syndrome but found no statistically significant differences in classification performance across the different groups (Figures S7 and S8, respectively).

### Feature importance

To assess the contribution of each feature to the classification task, we performed Shapley Additive exPlanations (SHAP) feature analysis on the best-performing ML model (XGBoost). SHAP displays class-specific effects of individual features on the model output [58, 59]. Thereby, the Shapley Value of a feature indicates its contribution to the difference between the model output and the average prediction.

**Figure 11.**
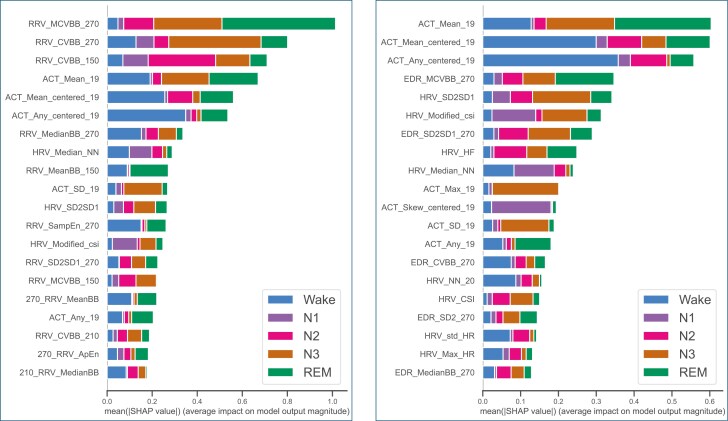
SHAP features importance (XGBoost) for input modality ACT + HRV + RRV (left) and ACT + HRV + ED-RRV (right).

Figure 11 displays the 20 features with the highest impact on the model prediction in Experiment 1 for the input modality combinations ACT + HRV + RRV and ACT + HRV + ED-RRV, respectively. Interestingly, the newly introduced RRV features appear to have the highest impact on the model output, indicated by the highest mean SHAP values. These features mainly contributed to the classification of sleep phases, while the movement-based features showed a higher influence on the classification of wake epochs. In general, introducing HRV features had a lower impact on the XGBoost model.

We had similar observations by using ACT + HRV + ED-RRV as input modality. However, the ED-RRV features showed generally a lower impact on the XGBoost model. We further observed that ACT features calculated on larger window sizes had a higher impact than those calculated on small sliding windows. The RRV and ED-RRV features showing the highest impact on the model output were time-domain features, while frequency-based (ED-)RRV features had lower SHAP values. HRV features from the time domain had a higher impact in the classification of wake epochs while the HRV features of frequency and non-linear domain showed a higher impact on distinguishing sleep epochs.

**Figure 12.**
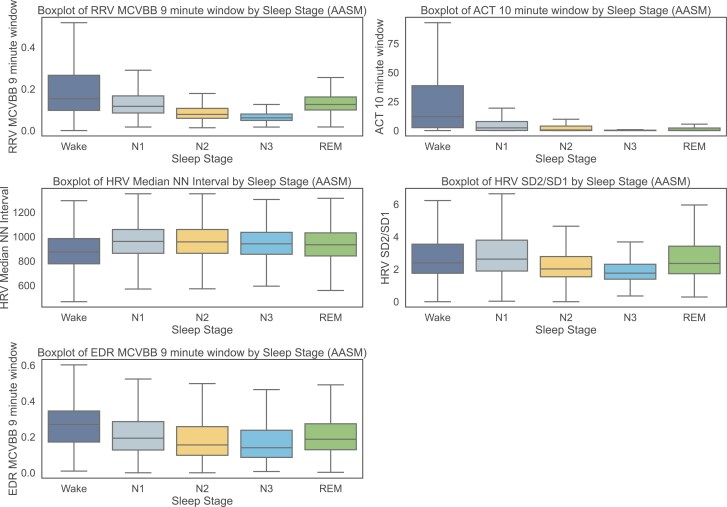
Features values per sleep stage of features with the highest SHAP value for sleep-stage classification according to AASM standards.

To assess the features from a physiological perspective, we analyzed the highest-ranked features in the SHAP analysis for each modality across specific sleep stages (Figure 12). The respiratory rate variability (RRV) and EDR feature MCVBB (measured over a 9-minute window) show higher values during wake and REM sleep epochs, compared to lower values observed in NREM phases, with the lowest values occurring in deep sleep. This trend was more prominent for the RRV feature. The ACT-based movement feature (ACT, calculated over a 10-minute window) demonstrated the highest values during wake states and progressively lower values across different sleep stages. For HRV, the median NN-interval feature revealed lower heart rates during sleep phases compared to wake states. Additionally, we observed low values for the SD2/SD1 feature during the N2 and N3 sleep phases and higher values for wake, N1, and REM sleep.

## Discussion

In this work, we present the first systematic comparison of sleep stage detection on a large benchmark dataset including ACT, HRV, and respiratory information collected from a respiration belt. We specifically investigate whether adding respiratory information to the established ACT + HRV-based classification enhances sleep stage detection. To avoid using additional respiratory sensors we also explored the extraction of respiratory information from ECG. We used two different DL and five different ML algorithms to classify sleep stages at different levels of granularity, ranging from wake/N1/N2/N3/REM classification, to wake/NREM/REM detection, and sleep–wake detection.

### General discussion

To systematically investigate the contribution of different modeling paradigms to sleep-stage classification, we included seven different algorithms from different algorithmic families. This also ensures comparability to previous work. Overall, the LSTM-based approach showed the best performance, outperforming all other combinations of input modalities across different levels of granularity in sleep stage classification. The best ML algorithm was XGBoost, with all ML algorithms performing within a similar range. Including cardiac information in the input modalities led to strong improvements for DL algorithms compared to using ACT-only, while ML algorithms showed only moderate improvements. These results are consistent with previous research [32].

Conversely, adding respiratory information to the ACT + HRV approach only led to a moderate increase in performance for the LSTM algorithm, while the ML algorithms showed a strong increase in performance. The larger performance boost while adding RRV to the ML algorithms might explain that the DL algorithms can inherently extract further information, for instance, frequency-based EDR, that the ML algorithms cannot do. However, as DL models are so-called black-box models [60], we cannot examine this in the current study, and this would need to be confirmed in future studies.

When we included respiratory information obtained from EDR instead of the respiratory belt, the performance of the ML algorithms only slightly increased compared to the ACT + HRV approach, and the DL algorithms did not consistently show better performance. This is particularly interesting because a proper extraction of EDR should result in ED-RRV features with similar information as contained in the RRV features. This indicates that the extraction of EDR by computing the mean amplitude of troughs and proceeding peaks [46] is not robust in all cases. Noise or other artifacts in the ECG signal, or variations in posture or cardiac behavior might affect the exactness of the EDR algorithm, potentially leading to a loss of critical respiratory information. As visible in Figure 2, the respiration signal computed via EDR contains amplitudes with higher variance as well as inhalation and exhalation peaks at slightly different locations. The lower detection rate of wake epochs by utilizing ED-RRV, compared to the approach using RRV, points out, that the EDR algorithm does not work accurately during wake phases. A possible reason is increased movement during wake phases that might distort the ECG signal leading to inaccurate respiration signal extracted via EDR. These inaccuracies can, in turn, impact downstream analyses, such as sleep stage estimation. To address this issue, future research should compare alternative algorithms for extracting respiratory information from ECG signals.

The feature importance analysis for the XGBoost models highlights the impact of respiratory information, particularly in subdividing REM and NREM sleep, while ACT-based features were most impactful in identifying wake periods. These findings suggest that combining multiple modalities could be advantageous. By analyzing the confusion matrices of the ML algorithms, it is visible that adding RRV features enables a better classification of Wake and REM sleep epochs (Figure 6). This is also visible for classification using ED-RRV features, however, to a lesser extent. A possible reason could be that respiration generally decreases during NREM sleep compared to wakefulness, while REM sleep is associated with more irregular breathing patterns [21]. In contrast, the DL algorithms already demonstrate good REM sleep detection using ACT and HRV. Adding RRV features primarily improves the classification of Wake epochs, which might explain the smaller boost in classification performance. This might indicate that the DL algorithms are more effective at capturing the relationship between HRV and distinct sleep phases compared to classical ML algorithms. Another possible reason is that the DL algorithms inherently learn respiratory characteristics from the HRV data as heart rate frequencies are influenced by respiration [40].

The classification framework presented by us enables sleep analysis using biosignals which can be obtained from consumer-grade devices. Thereby, the input features for HRV and RRV are based on the calculation of peak-to-peak variabilities. In order to make the process less obtrusive, heartbeat detection from ECG could potentially be replaced by analyzing PPG [7] from a smartwatch or by collecting cardiac information from ballistocardiography [61]. However, consumer-grade devices nowadays are mostly suitable for sleep–wake prediction only and suffer from sleep overprediction. Furthermore, those devices perform best on nights with consolidated sleep patterns, whereas in a medical context, the nights with abnormal sleep patterns would be more relevant [22].

In contrast to this work, previous work frequently extracted movement information from accelerometry [62, 63]. By analyzing 3-dimensional acceleration instead of ACT, more finely divided information about movements could be extracted such that more complex movement features could be extracted. Especially DL algorithms could benefit from including raw accelerometry in the classification process as they are able to inherently extract relevant features [63]. Furthermore, information about the movement of specific body parts or postures could be included in the classification process [64]. Furthermore, current research examines the contactless classification of sleep phases by extracting movement, respiratory, and cardiac information from data obtained by a CW Doppler radar mounted on the ceiling [29]. Moreover, current work shows promising results in classifying different sleep stages, detecting sleep apnea, and predicting the AHI using radar-based analysis [30].

### Sleep analysis by experiment

In the first experiment, we performed sleep-stage classification according to AASM standards where sleep phases are subdivided into wake/N1/N2/N3 and REM epochs. In general, classifying sleep stages into five classes according to AASM standards remains challenging, particularly when it comes to detecting non-REM epochs without EEG signals. Due to only small differences in movement, respiration, and heartbeats during these sleep phases, our algorithms were incapable of detecting N1 and N3 sleep at a reasonable rate. Class-imbalance is a challenge in sleep-staging, as certain sleep stages, such as N1 and N3 sleep, occur less frequently than N2 sleep. This imbalance was reflected in our results, as the majority of NREM epochs were classified as N2 sleep. One option to address this issue would be to weigh samples inversely proportional to class prevalence, allowing the model to put greater emphasis on underrepresented classes (Cost-sensitive learning). This technique can be used in large wearable sleep tracker datasets to improve the classification performance of minority classes. Additionally, techniques such as oversampling minority classes could be explored in future studies to improve classification performance for rarer sleep stages. Methods like SMOTE [65] (Synthetic Minority Over-sampling Technique) could be applied in hospital-based sleep monitoring systems to ensure better detection of minority classes in small-sized datasets. Moreover, while MCC was used as a robust evaluation metric to account for imbalanced data distributions, it is not inherently correct for class imbalance. Future work should integrate both metric-based and algorithmic-based approaches to class imbalance handling to ensure a more balanced learning process across all sleep stages. Nonetheless, about 50% correctly classified REM sleep epochs using ML algorithms a strong improvement compared to existing literature [32]. Depending on the medical use case, it might be sufficient for an initial screening to detect only NREM, REM, and wake stages, simplifying the classification process and still providing valuable insights for clinical evaluation.

As the detection of the single NREM phases was difficult in the first experiment, we aimed to distinguish between wake, NREM (combining N1, N2, and N3 sleep epochs), and REM epochs in the second experiment. Overall, the results were consistent with the first experiment, but with improved performance due to the combination of the single NREM sleep phases.

Within the third experiment, we performed sleep–wake detection. Traditionally, sleep analysis using unobtrusive sensors was mostly ACT-based using heuristic algorithms. Palotti et al. [31] examined ACT-based sleep–wake detection using heuristic, ML, and DL algorithms. As they found ML and DL algorithms both outperforming the heuristic algorithms these algorithms were not considered in this work. Nevertheless, five commonly used heuristic algorithms are implemented in the repository published in this paper and can be applied to the MESA-Sleep dataset as well ^4^. In contrast to the work of Zhai et al. [32] we found a significant increase in sleep–wake detection performance by introducing HRV features to the DL algorithms. Moreover, incorporating RRV features further enhanced the classification performance of DL and especially ML algorithms. These effects are explainable with a higher detection rate of wake epochs (+2.4% and +5.4%) which emphasizes that introducing further biosignals as input modalities successfully tackles the problem of sleep overprediction.

We found that the ACT-only approach performed worst in all experiments which is in line with previous investigations. This is often due to the overprediction of sleep which is a common problem in sleep analysis using movement-based sensor modalities. The reason for that might be a lack of movement shortly before falling asleep or in short periods of wakefulness [66]. Introducing further biosignals such as HRV or RRV might be able to tackle this problem as respiration rate, as well as cardiac activity, change concurrently with falling asleep and within different sleep phases [17, 18].

### Feature selection and validation

To further validate the physiological relevance of the features used in this study, we analyzed the most important features from each modality, as identified by SHAP analysis, and examined their behavior across different sleep stages.

Respiration is a physiological process influenced by autonomic control, and its variability provides insights into sleep stage transitions [19]. NREM sleep typically exhibits stable, regular breathing patterns, while REM sleep is characterized by irregular respiration [19, 21]. The RRV-MCVBB feature, computed over a centered 9-minute window, emerged as the most significant contributor in the SHAP analysis. This feature normalizes absolute variability (MaDBB) by the median breath interval (MedianBB), effectively capturing respiratory instability during REM sleep and differentiating it from more stable breathing in NREM sleep (Figure 12). The higher MCVBB values during the Wake state probably contribute to fluctuating activities. Analyzing the EDR-MCVBB feature revealed a similar trend to the RRV MCVBB feature. However, the variation between the sleep stages is only visible to a lower extend compared to the RRV feature, suggesting that while EDR can capture respiratory patterns, direct respiratory signals remain more accurate when available.

The (ED)-RRV features were selected based on the work of Soni et al. and implemented using the well-established python library Neurokit2 [50, 51]. This library provides a variety of RRV indices, including time-domain, frequency-domain, and nonlinear features. Within the power spectral analyses, different frequency bands were extracted and analyzed. However, the RRV HF band typically corresponds to variability in the 0.15 to 0.4 Hz range (equivalent to 2.5 to 7 seconds or 1–2 breathing cycles). Typically, breathing patterns in humans have slower dynamics. This aligns with the low feature attribution observed in the SHAP analysis. Similarly, the LFHF feature, while widely used in HRV analysis, may not capture meaningful respiratory mechanisms linked to sleep stages. Despite this, we included these features in our analysis to comprehensively evaluate the feature space. Future work could consider omitting features with limited physiological relevance.

HRV is a widely recognized indicator of autonomic nervous system activity and has been extensively studied in sleep research [20]. NREM sleep is associated with increased vagal control and more stable HRV, while REM sleep exhibits greater HRV variability due to sympathetic activation [20]. Our SHAP analysis ranked the median NN-interval as the most important HRV-based feature, likely due to its strong association with sleep stage-dependent heart rate regulation (especially lower heart rates during NREM periods, Figure 12). Additionally, the SD1/SD2 ratio was ranked highest in the EDR classification approach. This feature represents the balance between short-term and long-term variability in the heart’s inter-beat intervals. It is particularly relevant for capturing autonomic shifts during different sleep stages: NREM sleep is associated with lower SD2/SD1 ratios due to lower HRV [20], while REM sleep exhibits higher SD2/SD1 ratios due to irregular long-term HRV dynamics [20](Figure 12).

Movement-based features derived from actigraphy are well-established in sleep staging [10–14]. The ACT-based feature that contributed most to the SHAP analysis was ACT-Mean within a 10-minute epoch. As visible in the SHAP analysis, this feature was most effective in classifying wake epochs, which can also be seen by the largest deviation in the detailed feature analysis (Figure 12). This is expected, as actigraphy primarily captures motion-related differences, making it less effective in distinguishing sleep stages beyond wake versus sleep classification.

### Influences on classification performance

We conducted several statistical tests to assess the robustness of our sleep classification framework and determine whether participant characteristics such as age, gender, origin, or diseases affected classification performance. In contrast to the work of Vallat et al. [67], who reported slightly worse performance in elderly individuals, we found no influence of age on classification performance. However, their work was based on EEG features. They explain their findings with a higher proportion of N1 sleep typically seen in aging. We might not see these effects as our algorithm did not perform well in classifying N1 sleep. Analyzing the classification performance for individuals with high apnoe–hypopnoe index, we observed a decrease compared to individuals with low apnoe–hypopnoe index (Figure 10). These results are consistent with the work by Vallat et al. [67], who suspected that this might be driven by an increased number of sleep stage- and sleep–wake transitions. However, this decrease was larger when including cardiac- or respiratory information than with the classification of ACT-only. Possible reasons for that could be disturbed breathing due to apnea and hypopnea events during the night, which can have an impact on the cardiovascular system [68], possibly influencing the classification performance.

It is important to note that these findings are preliminary, as only a small subset of participants in the study were diagnosed with high apnea–hypopnea index. Therefore, future research needs to be conducted to confirm these findings.

To further investigate the robustness of our proposed framework, we examined the influence of gender, origin, and the diagnosis of insomnia or restless legs syndrome on classification performance. Our analysis revealed no significant differences between these groups, indicating that our approach for sleep staging is robust and can be applied to individuals with various backgrounds. However, respiratory or cardiovascular diseases might have an impact on classification performance.

### Limitations and future directions

While our approach made use of relatively simple out-of-the-box DL models, future research could focus on more complex and deeper models, such as ensembles or hybrid architectures, to potentially further enhance classification performance.

Another possibility to enhance the proposed classification framework is to incorporate prior knowledge about sleep physiology. A well-known characteristic of sleep is its cyclic nature, where REM sleep phases occur at roughly 90-minute intervals [47]. Incorporating such prior knowledge into our models could further enhance sleep classification performance. However, it might lead to weaker classification performance for individuals with irregular sleep patterns which might decrease the medical value.

The MESA sleep dataset is a large study that includes individuals from different ethnicities. However, one limitation of the MESA dataset is that the dataset was collected in a controlled laboratory, whilst the possible applications of sleep stage detection via wearable sensors are in real-world environments. This environment led to high data quality, but sleep habits and sleep quality might have been affected, which is why some conclusions are not completely applicable to real-world scenarios.

Contrary to the work of Vallet et al. who found a small decrease in classification performance for higher ages we did not find any age-related bias (Figure 9, Section 5.4). One possible reason for this is that the dataset consists predominantly of older adults (age range 45–85, median age 68.5 years) while Vallat et al. also included children and young adults in their analysis. This demographic distribution might introduce potential limitations regarding the generalizability of our findings to younger populations. While sleep behavior undergoes change across the lifespan, also factors like medication use and cardiovascular health may influence sleep or the physiological signals used for sleep stage classification. To account for potential age-related differences corrective measures such as age stratification could be applied. The model should then be subsequently tested on data from a younger cohort.

Additionally, while we assessed between-subject variability, within-subject variability could not be analyzed as the dataset contains only a signal night per participant. Using other datasets with multiple nights from the same individuals could further explore within-subject variability and its effects on classification performance. Furthermore, the framework was solely trained and tested on one dataset. To ensure the generalizability of the framework, it would be necessary to apply the models trained on the MESA sleep dataset to another dataset, which might also contain ACT with concurrent PSG recordings from different manufacturers.

## Conclusion and Outlook

Within this work, we compared the performance of seven commonly used state-of-the-art ML and DL algorithms. We observed that DL algorithms and, especially, the LSTM outperformed all other algorithms tested. Future work should therefore assess new model architectures with respect to classification performance, to further improve sleep analysis in a multistage classification setting and to make unobtrusive and wearable sleep analysis usable in a medical context. Our work demonstrates that including respiratory information in the already-established combination of ACT and HRV leads to significantly improved sleep stage classification. The ML algorithms profited most from introducing respiratory information, while the DL algorithms benefited most from adding cardiac information and were further enhanced by incorporating respiratory information. This underscores the great potential of respiratory information in unobtrusive sleep analysis.

Using ED-RRV features instead of RRV features we could only find smaller improvements in ML algorithms compared to the incorporation of respiratory information obtained from a respiration belt. Future work could therefore focus on developing more accurate algorithms to extract respiratory information from ECG data. Additionally, the cardiac or respiratory information used within this work could be collected less obtrusively or even contactless using different sensor technologies such as PPG or radar sensing. These alternatives would not only reduce discomfort for users but also have the potential to improve the quality of sleep data collection while also getting higher sleep data quality. By enabling long-term, continuous monitoring over weeks or months, these technologies provide deeper insights into sleep patterns, trends, and potential health risks, offering a more comprehensive view than traditional short-term, lab-based studies.

While this study focusses on the potential impact of respiratory information on sleep stage classification, other physiological parameters might also benefit sleep stage classification. For instance, future research could assess whether limb muscle activity measured via electromyography, or changes in electrodermal activity can give additional information. Furthermore, using accelerometry instead of actigraphy would allow us to get a more finely divided representation of movements during sleep which might benefit classification performance.

As most application scenarios of sleep analysis via wearable sensors are in real-world environments, future research should focus on collecting more real-world data resulting in larger datasets that can be used to systematically benchmark in a more realistic scenario to get more generalizable results.

With this work, we hope to contribute to the establishment of unobtrusive sleep analysis and encourage others to build upon our results. We hope that the availability of our code will encourage future research in the field of sleep-stage classification and contribute to the development of more accurate and reliable algorithms. Enabling accurate sleep assessment in home-monitoring environments will open the possibility of performing long-term sleep monitoring in a realistic setting, which can give us more insights into the prevention, diagnosis, and treatment of sleep disorders.

## Supplementary Material

zsaf091_suppl_Supplementary_Tables_S1-S8_Figures_S1-S8

## Data Availability

We conducted all experiments on data from the MESA-Sleep dataset, which is publicly available at https://sleepdata.org/datasets/mesa [41, 42].

## Footnotes

1
https://sleepdata.org/datasets/mesa/pages

2
https://github.com/Aura-healthcare/hrv-analysis

3
https://github.com/mad-lab-fau/tpcp

4
https://github.com/mad-lab-fau/sleep_analysis
