# Supplementary material for: Incorporating respiratory signals for machine learning-based multimodal sleep stage classification: a large-scale benchmark study with actigraphy and heart rate variability
Source: Sleep. 2025 Apr 11;48(9):zsaf091. doi: 10.1093/sleep/zsaf091 (PMC12417017; doi:10.1093/sleep/zsaf091)
Supplement: zsaf091_suppl_Supplementary_Tables_S1-S8_Figures_S1-S8 [file zsaf091_suppl_supplementary_tables_s1-s8_figures_s1-s8.zip › Sleep_Stage_Classification_large_dataset_supplementary_material/Sleep_Stage_Classification_large_dataset_supplementary_material.docx]

Incorporating Respiratory Signals for ML-based Multi-Modal Sleep Stage Classification: A Large-Scale Benchmark Study with Actigraphy and HRV

Daniel Krauss^1^, Robert Richer^1^, Arne Küderle^1^, Jelena Jukic^2^, Alexander German^2^, Heike Leutheuser^1^, Martin Regensburger^2^, Jürgen Winkler^2^, Bjoern M. Eskofier^1^

^1^Friedrich-Alexander-Universität (FAU) Erlangen-Nürnberg, Machine Learning and Data Analytics Lab

^2^Universitätsklinikum Erlangen, Department of Molecular Neurology

**Supplementary Material**

Corresponding Author:
Daniel Krauss

[Daniel.k.krauss@fau.de](mailto:Daniel.k.krauss@fau.de)

Carl Thiersch Strasse 2b

91052 Erlangen

Germany

**Supplementary Material**

**Supplementary Figure list:**

**Figure S1:** Visualization of the LSTM model architecture

**Figure S2:** Visualization of the TCN model architecture^70^

**Figure S3:** Confusion-matrix of sleep staging via LSTM classification into three stages (Wake / NREM/ REM). Values are given in %.

**Figure S4:** Confusion-matrix of sleep staging via XGBoost classification into three stages (Wake / NREM/ REM). Values are given in %.

**Figure S5:** Confusion-matrix of sleep/wake classification via LSTM.
Values are given in %.

**Figure S6:** Confusion-matrix of sleep/wake classification via XGBoost.
Values are given in %.

**Figure S7:** Influence of the diagnosis of the Restless Legs Syndrome on classification performance. *p < 0.05, **p < 0.01, ***p < 0.001.

**Figure S8:** Influence of the diagnosis of Insomnia on classification performance. *p < 0.05, **p < 0.01, ***p <0.001.

**Supplementary Tables**

*Table S1 Time-series features extracted from raw actigraphy (ACT). The features are extracted with varying sliding windows of duration 𝑡 and overlap of 𝑡 – 30 s.*

| Feature Name | Feature Description | Window size t in min |
| --- | --- | --- |
| ACC | Raw ACT count | 0.5 |
| LOG | Natural logarithm of activity count | 0.5 |
| Mean | Mean value of activity counts | 0.5 ≤ 𝑡 < 10 |
| Median | Median value of activity counts | 0.5 ≤ t < 10 |
| SD | Standard deviation of activity counts | 0.5 ≤ 𝑡 < 10 |
| Maximum | Maximum of activity counts | 0.5 ≤ 𝑡 < 10 |
| Minimum | Minimum of activity counts | 0.5 ≤ 𝑡 < 10 |
| Variance | Variance of activity counts | 0.5 ≤ 𝑡 < 10 |
| NAT | Number of epochs with activity counts larger than 50, but lower than 100 | 0.5 ≤ 𝑡 < 10 |
| ANY | Number of epochs that contain any activity count larger than 0 | 0.5 ≤ 𝑡 < 10 |
| Skewness | Skewness of ACT signal | 2 ≤ 𝑡 < 10 |
| Kurtosis | Kurtosis of ACT signal | 2 ≤ 𝑡 < 10 |

*Table S2 Heart rate variability (HRV) features extracted from NN-intervals grouped according to feature domain. The features are extracted from time-, geometrical-, frequency-, and non-linear domain in windows of 30 s.*

| Feature Name | Feature Description |
| --- | --- |
| Time-domain features | |
| Mean NN | Mean over NN-intervals |
| SDNN | Standard deviation of NN-intervals |
| SDSD | Standard deviation of NN differences |
| NN50 | Number of NN- Intervals greater than 50~ms |
| pNN50 | Ratio between NN50 and number of NN-intervals |
| NN20 | Number of NN- Intervals greater than 20~ms |
| pNN20 | Ratio between NN20 and number of NN-intervals |
| RMSSD | Root mean square of successive differences between NN-intervals |
| Median NN | Median of NN-intervals |
| Range NN | Range between smallest and largest NN-interval |
| CVSD | RMSSD divided by Mean NN (variation of successive differences) |
| CV NNI | The ratio of SDNN divided by Mean NN (variation of NN-intervals) |
| Mean HR | Mean Heart Rate |
| Max HR | Maximum Heart Rate |
| Min HR | Minimum Heart Rate |
| Std HR | Standard deviation of Heart Rate |
| Geometrical-domain features | |
| Triangular Index | Integral of density distribution of NN-intervals (number of all NN-intervals) divided by the maximum of the density distribution |
| Frequency-domain features | |
| LF | Variance (power) in low frequency (0.04 to 0.15 Hz) |
| HF | Variance (power) in high frequency (0.15 to 0.4 Hz) |
| VLF | Variance (power) in very low frequency (0.003 to 0.04 Hz) |
| LH/HF ratio | Ratio of low frequency to high frequency |
| LF norm | Normalized LF power |
| HF norm | Normalized HF power |
| Total Power | Total power |
| Non-linear domain features | |
| CSI | Cardiac Sympathetic Index^71^ |
| CVI | Cardiac Vagal Index^71^ |
| Modified CSI | Alternative measure of Cardiac Sympathetic Index^71^ |
| SD1 | The standard deviation of the projection of the Poincare plot^72^ |
| SD2 | SD2 is defined as the standard deviation of the projection of the Poincare plot on the line of identity^72^ |
| SD2SD1 | Ratio between SD2 and SD1^72^ |

*Table S3 Feature set containing respiratory information grouped according to feature domain. RRV features were computed on respiration waves obtained from a thoracic belt, while ED-RRV features were computed on EDR signals derived from ECG data. The features are extracted from time-, frequency-, and non-linear domain in centered windows of 5, 7 and 9 min.*

| Feature | Description | Window Size in min |
| --- | --- | --- |
| MeanBB | Mean duration of the breath-to-breath intervals | [5, 7, 9] min |
| SDBB | Standard deviation of the breath-to-breath intervals | [5, 7, 9] min |
| SDSD | Standard deviation of the successive differences between adjacent breath-to-breath intervals | [5, 7, 9] min |
| CVBB | SDBB / MeanBB | [5, 7, 9] min |
| CVSD | RMSSD / MeanBB | [5, 7, 9] min |
| MedianBB | Median duration of the breath-to-breath intervals | [5, 7, 9] min |
| MadBB | Mean absolute deviation of the breath-to-breath intervals | [5, 7, 9] min |
| MCVBB | MaDBB / MedianBB | [5, 7, 9] min |
| VLF | Variance (power) in very low frequency (0 to 0.04 Hz) | [5, 7, 9] min |
| LF | Variance (power) in low frequency (0.04 to 0.15 Hz) | [5, 7, 9] min |
| HF | Variance (power) in high frequency (0.15 to 0.4 Hz) | [5, 7, 9] min |
| LH/HF ratio | Ratio of low frequency to high frequency | [5, 7, 9] min |
| LFn | Normalized low frequency, obtained by dividing the low frequency power by the total power | [5, 7, 9] min |
| HFn | Normalized high frequency, obtained by dividing the high frequency power by total power | [5, 7, 9] min |
| SD1 | Measure of the spread of breath-to-breath intervals on the Poincaré plot perpendicular to the line of identity. It is an index of short-term variability | [5, 7, 9] min |
| SD2 | Measure of the spread of breath-to-breath intervals on the Poincaré plot along to the line of identity. It is an index of long-term variability | [5, 7, 9] min |
| SD2SD1 | Ratio between SD2 and SD1 | [5, 7, 9] min |
| ApEn | Approximate Entropy of Respiration Rate Variability | [5, 7, 9] min |
| SampEn | Sample Entropy of Respiration Rate Variability | [5, 7, 9] min |
| RMSSD | Root mean square of successive differences of the breath-to-breath intervals | [5, 7, 9] min |

*Table S4 Hyperparameter search space including the best set of hyperparameters for the classical ML models*

| **Algorithm** | **Parameter** | **Range** | **Best Hyperparameter (Task 1, ACT + HRV + RRV)** |
| --- | --- | --- | --- |
| **SVM** | loss | [hinge, log, modified_huber] | log |
|  | penalty | [l1, l2] | l2 |
|  | alpha | [0.0001, 0.001, 0.01] | 0.0001 |
|  | learning_rate | [optimal, adaptive] | adaptive |
|  | power_t | [0.0, 1.0], stepsize = 0.1 | 0.5 |
|  | max_iter | [1000, 1500] | 1500 |
|  | early_stopping | [True, False] | False |
| **Random Forest** | n_estimators | [10, 350], stepsize = 10 | 350 |
|  | max_depth | [5, 40], stepsize = 1 | 21 |
|  | min_samples_split | [2, 70], stepsize = 1 | 25 |
|  | min_samples_leaf | [1, 50], stepsize = 1 | 3 |
|  | max_features | [sqrt, log2] | sqrt |
| **AdaBoost** | learning_rate | [0.1, 1.0, 1.5] | 1.0 |
|  | n_estimators | [10, 50, 100, 500] | 500 |
| **MLP** | hidden_layer_sizes | [(50, 50, 50), (50, 100, 50), (100,)] | -100 |
|  | activation | [tanh, relu] | relu |
|  | solver | [adam] | adam |
|  | alpha | [1e-4, 0.05] | 0.05 |
|  | learning_rate | [constant, adaptive] | adaptive |
| **XGBoost** | n_estimators | [200, 400], stepsize = 1 | 389 |
|  | max_depth | [5, 25], stepsize = 1 | 9 |
|  | reg_alpha | [0, 25], stepsize = 1 | 25 |
|  | reg_lambda | [0, 25], stepsize = 1 | 6 |
|  | min_child_weight | [0, 25], stepsize = 1 | 7 |
|  | gamma | [5, 25], stepsize = 1 | 20 |
|  | learning_rate | [0.01, 0.1], stepsize = 0.01 | 0.0850 |
|  | colsample_by_tree | [0.1, 1], stepsize = 0.1 | 0.9753 |

*Table S5 Hyperparameter search space including the best set of hyperparameters for the DL models*

| **Algorithm** | **Parameter** | **Range** | **Best Hyperparameter (Task 1, ACT + HRV + RRV)** |
| --- | --- | --- | --- |
| **LSTM** | sequence_length | [21, 51, 101] | 101 |
|  | num_layers | [1, 5], stepsize = 1 | 3 |
|  | learning_rate | [1e-4, 5e-3] stepsize = 0.0001 | 0.000358 |
|  | batch_size | [32, 64, 128, 256, 512] | 512 |
|  | hidden_size | [4, 600], stepsize = 4 | 288 |
| **TCN** | sequence_length | [21, 51, 101] | 101 |
|  | num_chanels | [2, 6], stepsize = 1 | 5 |
|  | n_hid | [8, 512], stepsize = 32 | 296 |
|  | kernel_size | [2, 5], stepsize = 1 | 4 |
|  | dropout | [0.1, 0.5], stepsize = 0.1 | 0.3 |
|  | learning_rate | [1e-4, 1], stepsize = 1e-4 | 0.001544 |
|  | batch_size | [64, 128, 256] | 256 |

*Table S6 Algorithm performances of sleep staging according to AASM guidelines (Wake / N1 / N2 / N3 / REM) dependent on input modality combination. ACT = Actigraphy, HRV = Heart rate variability, RRV = respiration-rate variability, ED-RRV = ECG-derived respiration-rate variability. Values were computed per participant and then aggregated as median (IQR) (interquartile range). DL and ML algorithms are separated by a line. The best performance per modality is written in bold.*

|  | Accuracy [%] | F1-score [%] | MCC | Precision [%] | Recall [%] | Specificity [%] |
| --- | --- | --- | --- | --- | --- | --- |
| ACT | | | | | | |
| LSTM | **56.1 (13.1)** | **46.5 (13.2)** | **0.37 (0.18)** | **45.5 (14.9)** | **56.1 (13.1)** | **86.8 (08.4)** |
| TCN | 56.1 (13.3) | 46.8 (13.2) | 0.37 (0.18) | 45.5 (14.1) | 56.1 (13.3) | 86.5 (08.0) |
| AdaBoost | 55.0 (14.1) | 45.9 (14.3) | 0.33 (0.19) | 44.1 (15.2) | 55.0 (14.1) | 85.6 (08.3) |
| MLP | 54.9 (13.5) | 46.2 (13.6) | 0.34 (0.18) | 43.6 (14.6) | 54.9 (13.5) | 85.0 (08.3) |
| Random Forest | 55.1 (13.6) | 45.6 (13.7) | 0.34 (0.19) | 43.6 (13.8) | 55.1 (13.6) | 85.3 (08.3) |
| SVM | 55.0 (14.5) | 45.8 (14.8) | 0.33 (0.19) | 43.8 (14.5) | 55.0 (14.5) | 85.6 (09.3) |
| XGBoost | 55.2 (14.3) | 45.9 (14.4) | 0.34 (0.19) | 43.8 (14.7) | 55.2 (14.3) | 85.2 (08.2) |
| ACT + HRV | | | | | | |
| LSTM | **64.9 (14.9)** | **59.6 (15.5)** | **0.50 (0.17)** | **65.8 (13.8)** | **64.9 (14.9)** | **88.4 (06.5)** |
| TCN | 61.4 (12.5) | 55.3 (13.6) | 0.44 (0.16) | 60.5 (14.5) | 61.4 (12.5) | 86.5 (07.4) |
| AdaBoost | 56.4 (12.5) | 48.7 (12.3) | 0.36 (0.16) | 52.8 (14.0) | 56.4 (12.5) | 85.5 (07.1) |
| MLP | 56.8 (12.3) | 48.6 (13.2) | 0.36 (0.16) | 53.6 (13.1) | 56.8 (12.3) | 85.6 (07.5) |
| Random Forest | 56.9 (12.5) | 48.5 (13.1) | 0.36 (0.17) | 52.0 (14.8) | 56.9 (12.5) | 85.6 (07.4) |
| SVM | 56.3 (12.6) | 47.0 (13.2) | 0.35 (0.16) | 47.5 (14.2) | 56.3 (12.6) | 85.6 (07.9) |
| XGBoost | 57.2 (12.0) | 49.3 (12.1) | 0.37 (0.17) | 53.4 (13.8) | 57.2 (12.0) | 86.0 (06.6) |
| ACT + HRV + RRV | | | | | | |
| LSTM | **65.7 (11.9)** | **60.1 (12.4)** | **0.51 (0.16)** | **64.6 (13.5)** | **65.7 (11.9)** | **90.2 (06.3)** |
| TCN | 62.4 (12.7) | 57.9 (13.1) | 0.46 (0.18) | 63.0 (14.8) | 62.4 (12.7) | 87.1 (06.8) |
| AdaBoost | 61.9 (12.1) | 56.7 (12.9) | 0.44 (0.17) | 61.1 (13.6) | 61.9 (12.1) | 86.1 (07.2) |
| MLP | 62.7 (12.4) | 56.6 (13.5) | 0.46 (0.15) | 58.5 (11.3) | 62.7 (12.4) | 87.9 (07.2) |
| Random Forest | 62.3 (12.4) | 55.7 (12.6) | 0.45 (0.17) | 58.7 (13.8) | 62.3 (12.4) | 87.7 (07.2) |
| SVM | 60.5 (12.1) | 53.4 (12.9) | 0.42 (0.16) | 57.8 (14.5) | 60.5 (12.1) | 87.7 (07.8) |
| XGBoost | 63.2 (12.0) | 57.2 (12.8) | 0.46 (0.15) | 60.1 (13.1) | 63.2 (12.0) | 87.5 (07.3) |
| ACT + HRV + ED-RRV | | | | | | |
| LSTM | **63.7 (13.1)** | **57.2 (14.8)** | **0.49 (0.16)** | 60.9 (13.9) | **63.7 (13.1)** | **88.9 (06.3)** |
| TCN | 62.0 (12.6) | 56.6 (14.6) | 0.46 (0.18) | **62.1 (14.2)** | 62.0 (12.6) | 86.5 (07.3) |
| AdaBoost | 57.2 (12.2) | 50.5 (13.0) | 0.37 (0.15) | 55.4 (17.2) | 57.2 (12.2) | 85.4 (07.7) |
| MLP | 57.9 (13.0) | 50.9 (13.9) | 0.38 (0.16) | 56.0 (14.7) | 57.9 (13.0) | 85.4 (07.4) |
| Random Forest | 57.5 (12.8) | 50.0 (14.1) | 0.38 (0.17) | 53.4 (16.5) | 57.5 (12.8) | 86.3 (07.9) |
| SVM | 56.8 (12.2) | 48.3 (12.4) | 0.36 (0.16) | 52.1 (15.5) | 56.8 (12.2) | 85.5 (07.6) |
| XGBoost | 58.2 (12.2) | 51.1 (13.7) | 0.38 (0.16) | 55.1 (14.9) | 58.2 (12.2) | 86.1 (07.8) |

*Table S7 Algorithm performances for sleep staging into three stages (Wake / NREM / REM) dependent on input modality combination. ACT = Actigraphy, HRV = Heart rate variability, RRV = respiration-rate variability, ED-RRV = ECG-derived respiration respiration-rate variability. Values were computed per participant and then aggregated as median (IQR) (interquartile range). range. DL and ML algorithms are separated by a line. The best performance per modality is written in bold.*

|  | Accuracy [%] | F1-score [%] | MCC | Precision [%] | Recall [%] | Specificity [%] |
| --- | --- | --- | --- | --- | --- | --- |
| ACT | | | | | | |
| LSTM | **71.5 (11.4)** | **65.3 (11.7)** | **0.47 (0.21)** | **64.7 (11.1)** | **71.5 (11.4)** | **84.9 (12.5)** |
| TCN | 59.2 (09.4) | 53.1 (08.6) | 0.19 (0.16) | 51.1 (08.5) | 59.2 (09.4) | 67.5 (12.3) |
| AdaBoost | 70.0 (12.7) | 64.1 (13.7) | 0.44 (0.23) | 63.7 (10.6) | 70.0 (12.7) | 82.9 (13.2) |
| MLP | 70.1 (12.0) | 64.2 (13.7) | 0.44 (0.23) | 63.8 (10.2) | 70.1 (12.0) | 83.1 (12.5) |
| Random Forest | 70.1 (13.3) | 64.1 (13.8) | 0.44 (0.23) | 64.0 (10.5) | 70.1 (13.3) | 83.5 (13.2) |
| SVM | 69.8 (11.9) | 63.9 (13.9) | 0.44 (0.23) | 63.5 (11.0) | 69.8 (11.9) | 83.1 (12.2) |
| XGBoost | 70.3 (12.6) | 64.4 (13.8) | 0.45 (0.23) | 64.0 (10.3) | 70.3 (12.6) | 83.4 (12.9) |
| ACT + HRV | | | | | | |
| LSTM | **77.7 (10.7)** | **75.5 (12.2)** | **0.60 (0.19)** | **80.4 (09.2)** | **77.7 (10.7)** | **87.4 (08.2)** |
| TCN | 75.6 (12.4) | 73.3 (13.2) | 0.55 (0.21) | 77.1 (10.0) | 75.6 (12.4) | 84.0 (11.0) |
| AdaBoost | 70.6 (11.3) | 65.2 (12.8) | 0.45 (0.20) | 68.2 (11.9) | 70.6 (11.3) | 83.3 (12.3) |
| MLP | 70.9 (11.1) | 65.8 (11.8) | 0.46 (0.20) | 69.8 (11.8) | 70.9 (11.1) | 83.4 (12.0) |
| Random Forest | 71.2 (11.3) | 65.9 (12.3) | 0.46 (0.20) | 68.6 (13.0) | 71.2 (11.3) | 83.5 (12.2) |
| SVM | 70.2 (11.5) | 64.5 (12.9) | 0.44 (0.20) | 64.0 (09.7) | 70.2 (11.5) | 83.3 (12.4) |
| XGBoost | 71.0 (11.4) | 66.3 (11.6) | 0.46 (0.19) | 69.9 (12.1) | 71.0 (11.4) | 83.7 (12.7) |
| ACT + HRV + RRV | | | | | | |
| LSTM | **79.0 (09.8)** | **77.7 (11.3)** | **0.62 (0.20)** | **80.2 (09.8)** | **79.0 (09.8)** | **86.9 (09.9)** |
| TCN | 73.1 (10.2) | 70.6 (11.5) | 0.51 (0.19) | 74.4 (09.9) | 73.1 (10.2) | 82.9 (10.2) |
| AdaBoost | 74.1 (10.2) | 72.3 (11.4) | 0.53 (0.19) | 76.6 (09.1) | 74.1 (10.2) | 83.6 (11.2) |
| MLP | 75.6 (10.3) | 73.9 (11.3) | 0.55 (0.17) | 77.2 (08.1) | 75.6 (10.3) | 84.2 (09.5) |
| Random Forest | 74.5 (10.2) | 72.6 (12.3) | 0.55 (0.18) | 76.5 (09.2) | 74.5 (10.2) | 84.9 (10.1) |
| SVM | 72.7 (10.7) | 68.6 (11.6) | 0.50 (0.18) | 74.1 (11.4) | 72.7 (10.7) | 84.9 (12.4) |
| XGBoost | 75.5 (10.4) | 74.1 (11.7) | 0.56 (0.17) | 77.3 (08.5) | 75.5 (10.4) | 84.4 (10.7) |
| ACT + HRV + ED-RRV | | | | | | |
| LSTM | **77.5 (11.5)** | **75.6 (13.1)** | **0.59 (0.20)** | **79.8 (09.5)** | **77.5 (11.5)** | **87.3 (09.1)** |
| TCN | 71.4 (10.6) | 67.4 (11.5) | 0.48 (0.17) | 72.8 (11.4) | 71.4 (10.6) | 83.6 (10.2) |
| AdaBoost | 71.0 (11.0) | 66.2 (12.1) | 0.46 (0.20) | 71.3 (11.9) | 71.0 (11.0) | 83.2 (12.3) |
| MLP | 71.7 (11.4) | 67.3 (12.7) | 0.47 (0.20) | 70.5 (13.9) | 71.7 (11.4) | 83.0 (11.6) |
| Random Forest | 71.6 (11.5) | 66.8 (12.7) | 0.47 (0.21) | 71.2 (12.8) | 71.6 (11.5) | 83.5 (11.2) |
| SVM | 70.8 (10.9) | 65.4 (12.5) | 0.46 (0.20) | 67.2 (13.3) | 70.8 (10.9) | 83.3 (11.7) |
| XGBoost | 72.1 (11.1) | 67.7 (11.9) | 0.48 (0.20) | 72.6 (10.5) | 72.1 (11.1) | 83.3 (11.2) |

*Table S8 Algorithm performances sleep/wake classification dependent on input modality combination. ACT = Actigraphy, HRV = Heart rate variability, RRV = respiration-rate variability, ED-RRV = ECG-derived respiration respiration-rate variability. Values were computed per participant and then aggregated as median (IQR) (interquartile range). DL and ML algorithms are separated by a line. The best performance per modality is written in bold.*

|  | Accuracy [%] | F1-score [%] | MCC | Precision [%] | Recall [%] | Specificity [%] |
| --- | --- | --- | --- | --- | --- | --- |
| ACT | | | | | | |
| LSTM | **85.1 (13.2)** | 89.2 (10.7) | **0.62 (0.27)** | **88.7 (13.9)** | 94.3 (10.2) | **70.9 (33.3)** |
| TCN | 84.9 (14.2) | **89.5 (11.0)** | **0.62 (0.27)** | **88.7 (14.6)** | 94.1 (10.3) | 70.7 (32.5) |
| AdaBoost | 83.9 (14.2) | 89.2 (12.3) | 0.57 (0.29) | 85.1 (15.6) | 96.5 (09.0) | 60.1 (32.4) |
| MLP | 84.1 (13.8) | 89.2 (11.9) | 0.59 (0.28) | 84.7 (15.3) | 96.5 (08.1) | 60.4 (32.2) |
| Random Forest | 84.4 (14.2) | 89.0 (12.1) | 0.59 (0.28) | 84.9 (15.7) | 96.2 (08.4) | 60.9 (32.8) |
| SVM | 83.1 (13.9) | 88.8 (11.9) | 0.57 (0.27) | 84.5 (15.6) | **96.6 (08.1)** | 57.4 (32.7) |
| XGBoost | 84.2 (14.0) | 89.1 (12.2) | 0.58 (0.28) | 85.1 (16.2) | 96.3 (08.4) | 60.5 (33.3) |
| ACT + HRV | | | | | | |
| LSTM | **87.3 (10.3)** | **91.2 (09.2)** | **0.68 (0.25)** | **89.6 (13.4)** | 94.8 (06.7) | **76.4 (28.7)** |
| TCN | 84.9 (11.6) | 89.6 (10.5) | 0.61 (0.26) | 86.9 (14.5) | 95.5 (07.2) | 64.4 (28.5) |
| AdaBoost | 84.3 (14.1) | 89.2 (12.3) | 0.60 (0.26) | 86.1 (15.8) | 95.7 (10.0) | 64.8 (33.8) |
| MLP | 84.0 (13.8) | 89.4 (11.8) | 0.60 (0.26) | 86.6 (15.0) | 95.7 (08.9) | 66.1 (34.4) |
| Random Forest | 84.9 (14.1) | 89.5 (12.4) | 0.60 (0.27) | 86.1 (16.1) | 95.4 (08.6) | 64.4 (35.6) |
| SVM | 84.0 (13.8) | 89.2 (12.0) | 0.58 (0.25) | 86.0 (15.5) | **96.1 (09.2)** | 61.8 (34.5) |
| XGBoost | 84.7 (14.0) | 89.2 (11.9) | 0.61 (0.26) | 86.6 (15.8) | 95.3 (09.6) | 65.9 (33.5) |
| ACT + HRV + RRV | | | | | | |
| LSTM | **87.9 (09.3)** | **91.0 (08.5)** | **0.71 (0.22)** | **90.7 (12.3)** | 94.4 (07.3) | **76.9 (27.6)** |
| TCN | 86.3 (11.6) | 90.1 (09.7) | 0.66 (0.24) | 89.8 (12.9) | 93.1 (09.0) | 73.5 (26.5) |
| AdaBoost | 85.9 (13.0) | 90.1 (09.9) | 0.63 (0.25) | 87.2 (14.8) | 95.1 (08.1) | 69.4 (32.5) |
| MLP | 86.1 (11.6) | 90.1 (09.7) | 0.66 (0.22) | 88.3 (13.0) | 95.1 (07.1) | 71.9 (27.9) |
| Random Forest | 86.0 (13.2) | 90.1 (10.0) | 0.64 (0.24) | 87.6 (14.0) | 95.7 (07.1) | 68.0 (33.8) |
| SVM | 85.1 (12.8) | 89.9 (10.5) | 0.62 (0.24) | 86.9 (14.1) | **96.4 (08.0)** | 66.6 (33.2) |
| XGBoost | 86.4 (12.3) | 90.4 (09.6) | 0.66 (0.23) | 88.3 (13.5) | 95.4 (07.5) | 71.1 (28.7) |
| ACT + HRV + ED-RRV | | | | | | |
| LSTM | **86.5 (10.7)** | **90.1 (08.9)** | **0.67 (0.26)** | **89.0 (14.4)** | 94.1 (08.7) | **73.0 (26.6)** |
| TCN | 85.3 (12.2) | 89.9 (09.6) | 0.63 (0.23) | 87.0 (14.2) | 95.3 (07.1) | 67.2 (28.3) |
| AdaBoost | 84.3 (14.0) | 89.2 (12.0) | 0.60 (0.26) | 86.7 (13.8) | 95.5 (10.6) | 67.0 (34.3) |
| MLP | 84.9 (13.0) | 89.1 (11.6) | 0.62 (0.25) | 87.1 (14.6) | 95.4 (08.4) | 67.9 (34.3) |
| Random Forest | 85.1 (13.6) | 89.4 (12.0) | 0.61 (0.27) | 86.3 (15.2) | 95.4 (08.9) | 64.3 (34.7) |
| SVM | 84.2 (13.8) | 89.2 (11.8) | 0.60 (0.27) | 86.6 (14.3) | **95.8 (09.5)** | 64.1 (34.1) |
| XGBoost | 84.7 (13.5) | 89.2 (11.6) | 0.61 (0.26) | 87.0 (13.7) | 95.4 (09.4) | 66.4 (35.7) |
