# Supplementary figures and images for: Incorporating respiratory signals for machine learning-based multimodal sleep stage classification: a large-scale benchmark study with actigraphy and heart rate variability
Source: Sleep. 2025 Apr 11;48(9):zsaf091. doi: 10.1093/sleep/zsaf091 (PMC12417017; doi:10.1093/sleep/zsaf091)

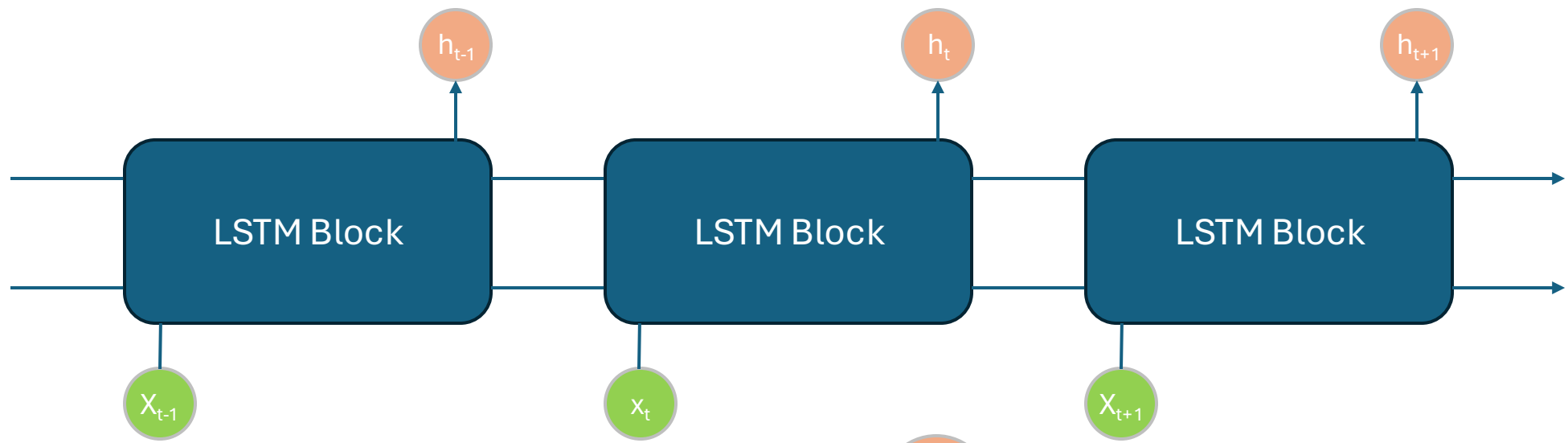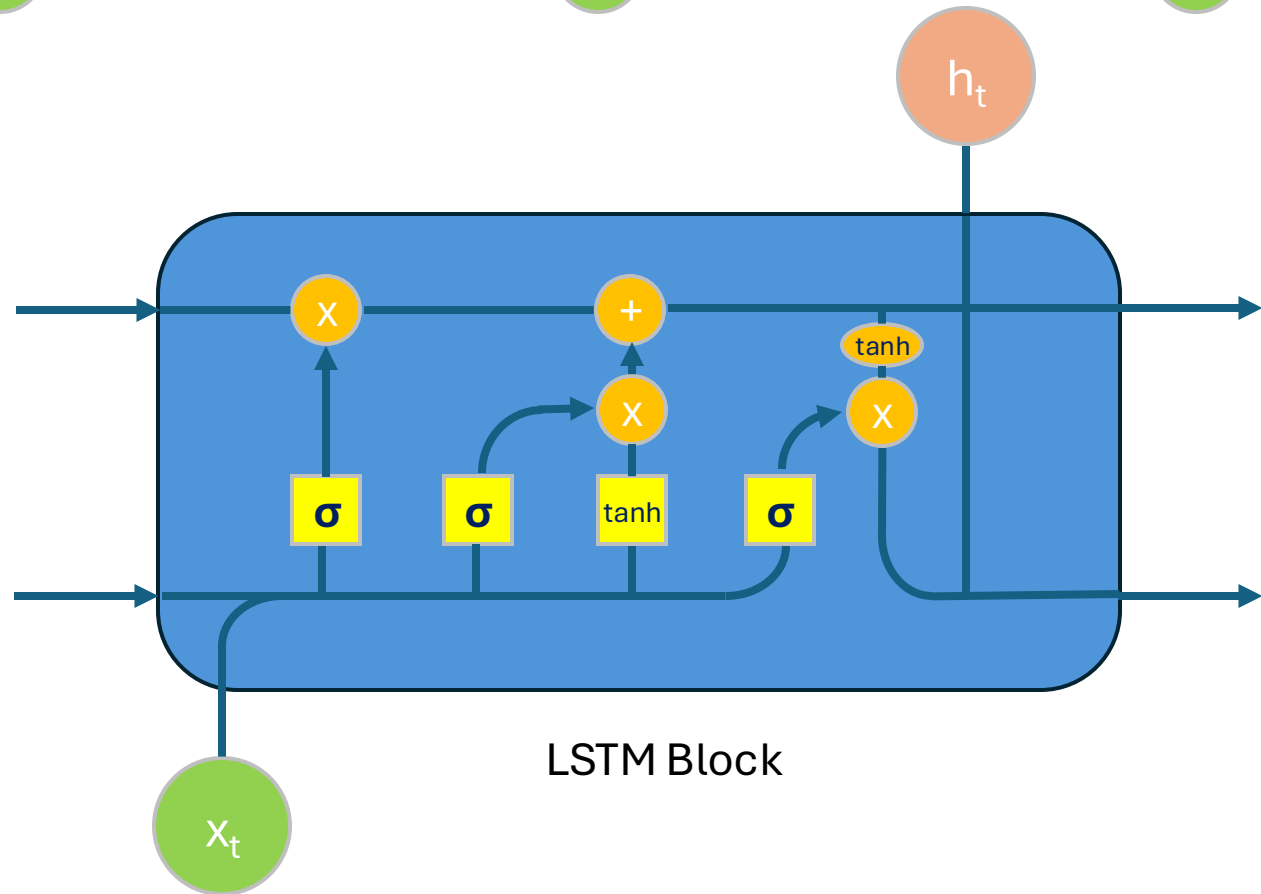

Supplement: zsaf091_suppl_Supplementary_Tables_S1-S8_Figures_S1-S8 [file zsaf091_suppl_supplementary_tables_s1-s8_figures_s1-s8.zip › Sleep_Stage_Classification_large_dataset_supplementary_material/Figure_S1_lstm.pdf]

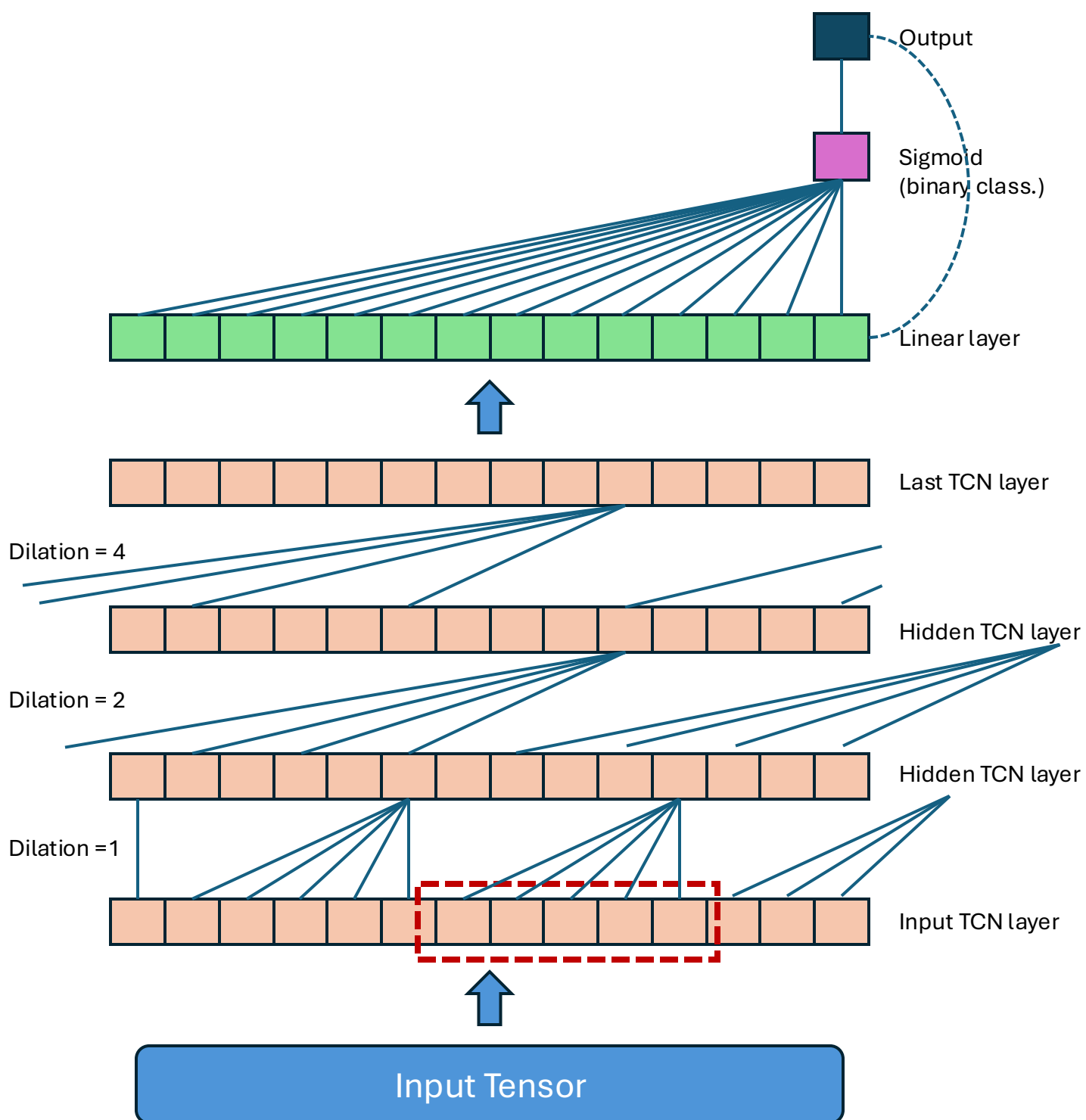

Supplement: zsaf091_suppl_Supplementary_Tables_S1-S8_Figures_S1-S8 [file zsaf091_suppl_supplementary_tables_s1-s8_figures_s1-s8.zip › Sleep_Stage_Classification_large_dataset_supplementary_material/Figure_S2_tcn.pdf]

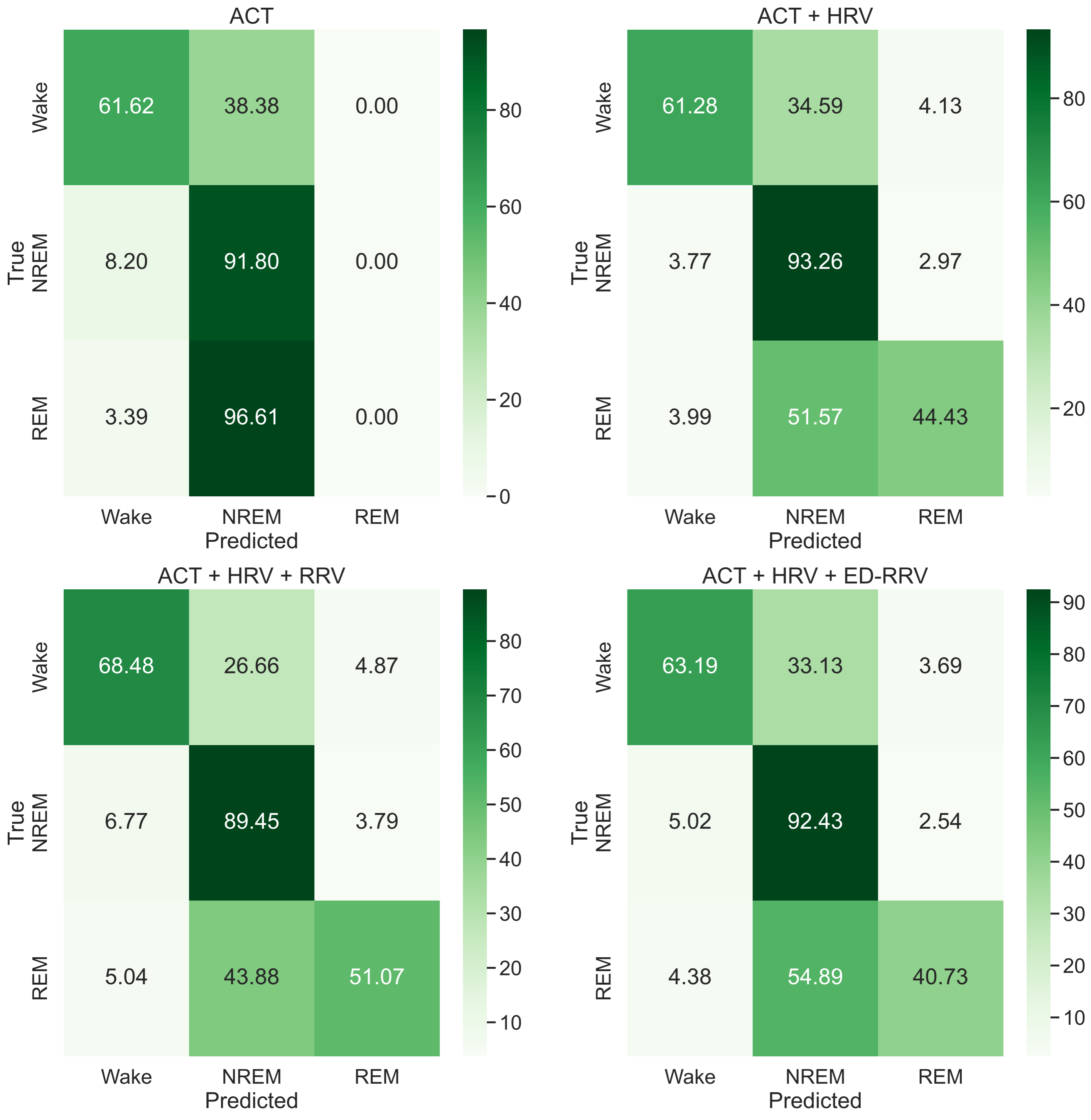

Supplement: zsaf091_suppl_Supplementary_Tables_S1-S8_Figures_S1-S8 [file zsaf091_suppl_supplementary_tables_s1-s8_figures_s1-s8.zip › Sleep_Stage_Classification_large_dataset_supplementary_material/Figure_S3_confusionmatrix_LSTM_3stage.pdf]

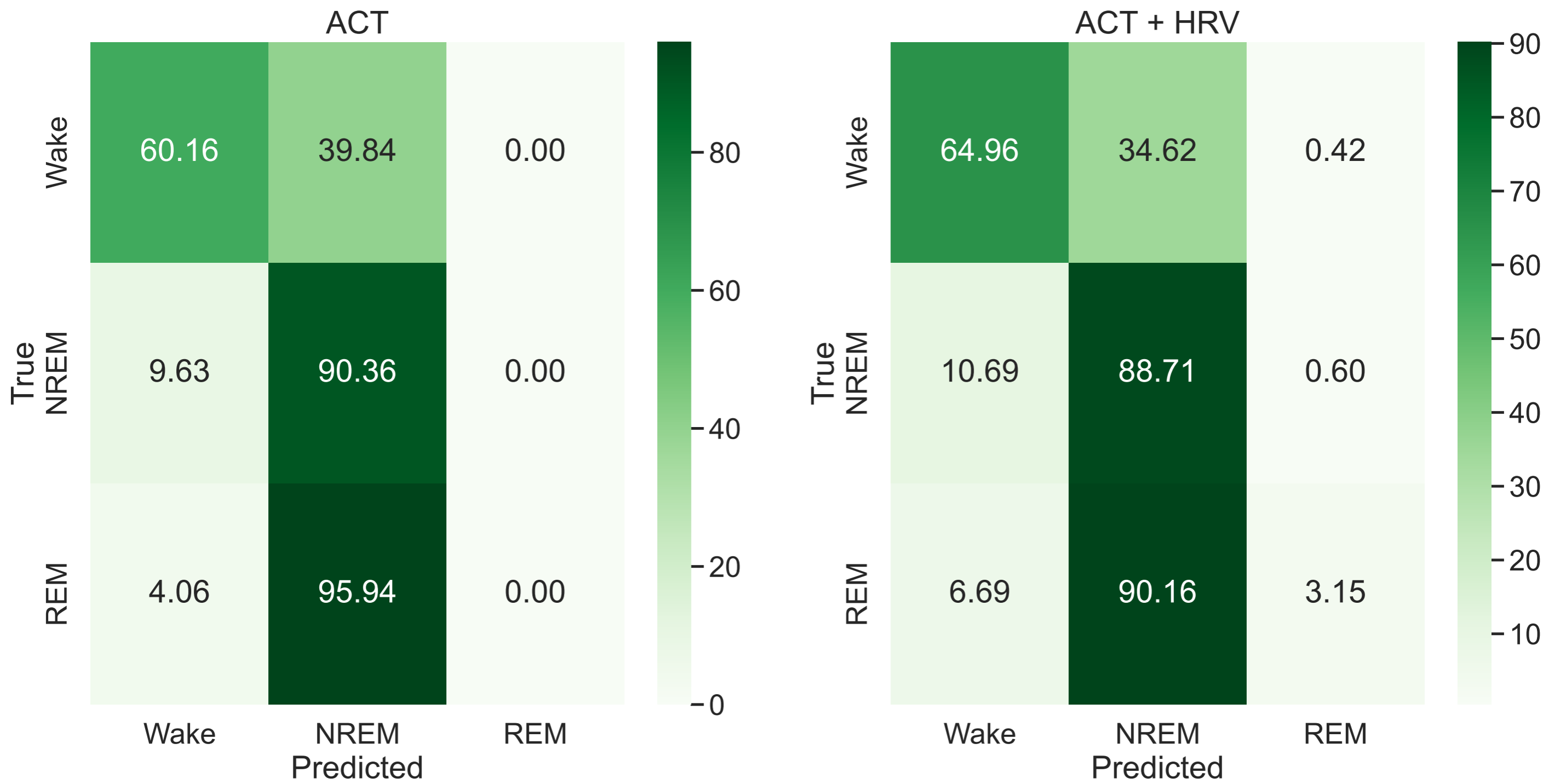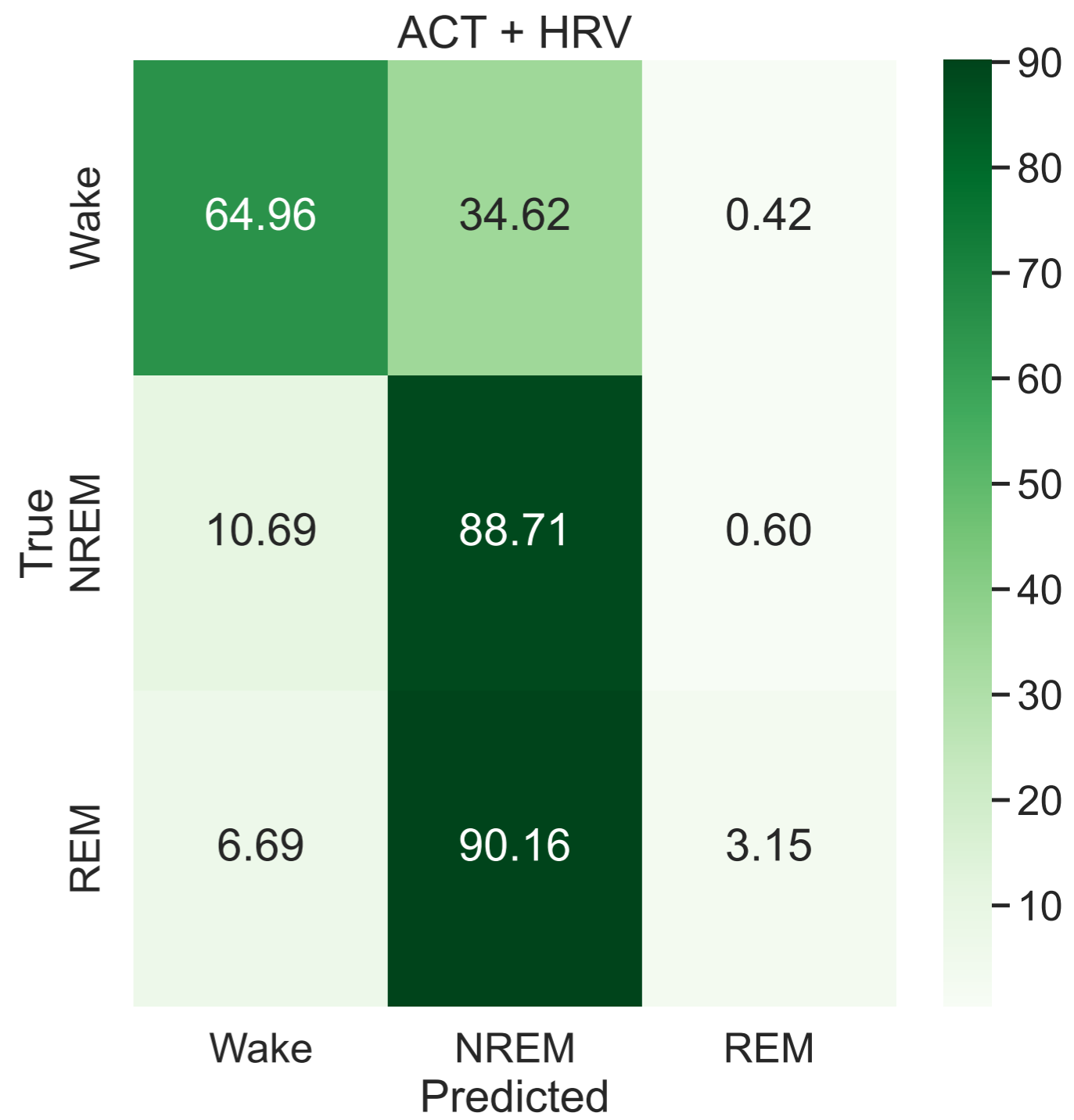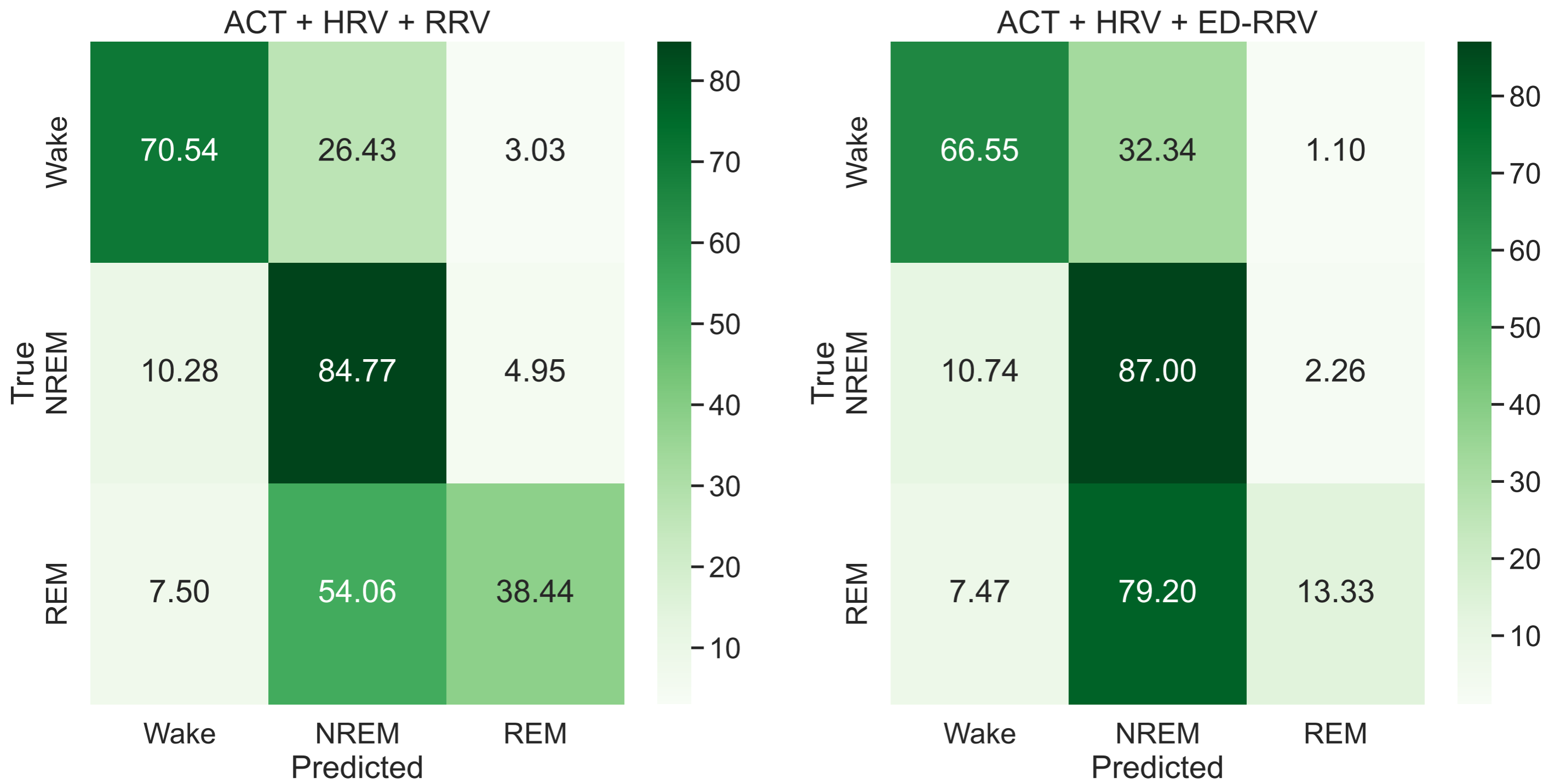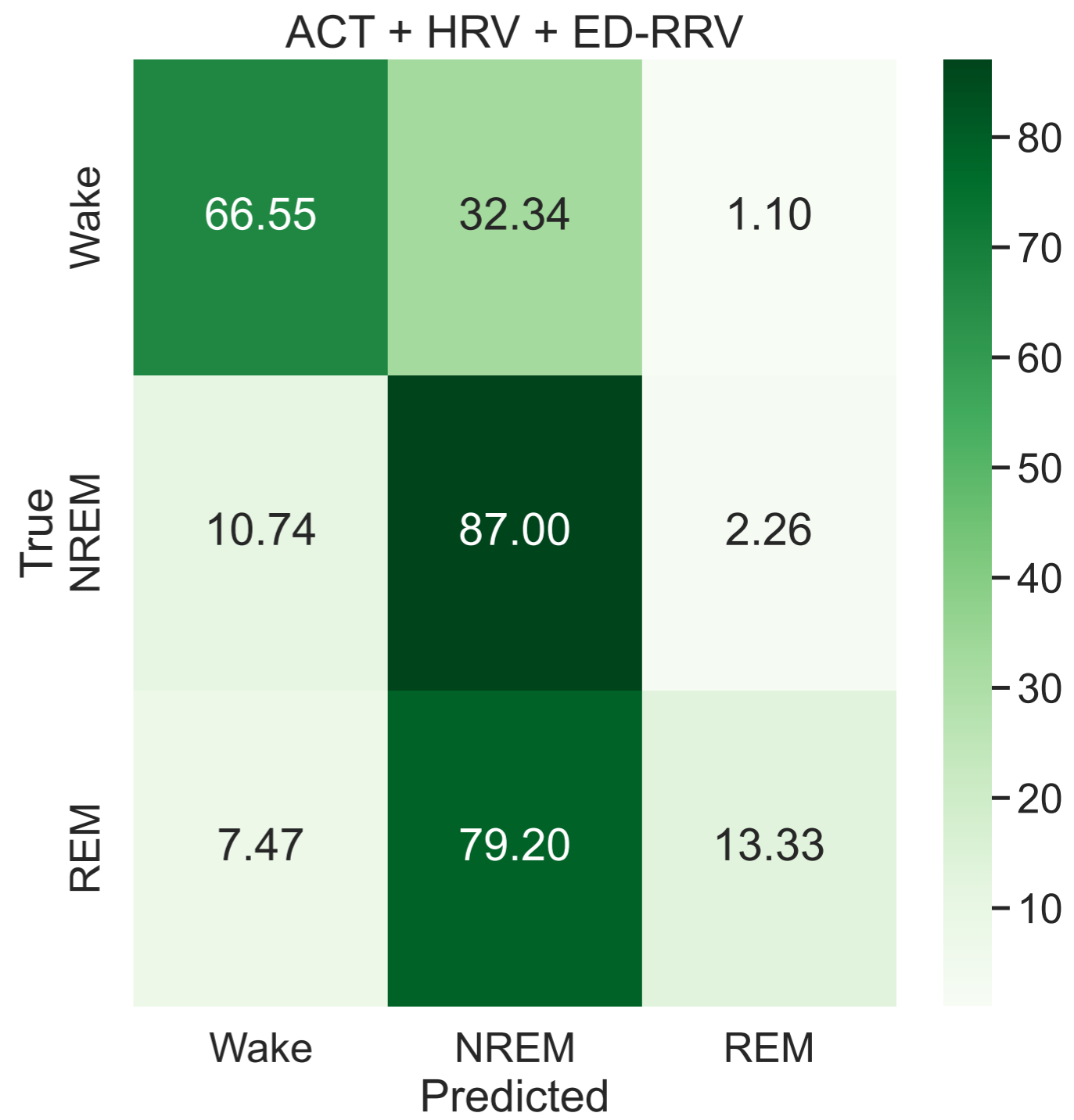

Supplement: zsaf091_suppl_Supplementary_Tables_S1-S8_Figures_S1-S8 [file zsaf091_suppl_supplementary_tables_s1-s8_figures_s1-s8.zip › Sleep_Stage_Classification_large_dataset_supplementary_material/Figure_S4_confusionmatrix_xgb_3stage.pdf]

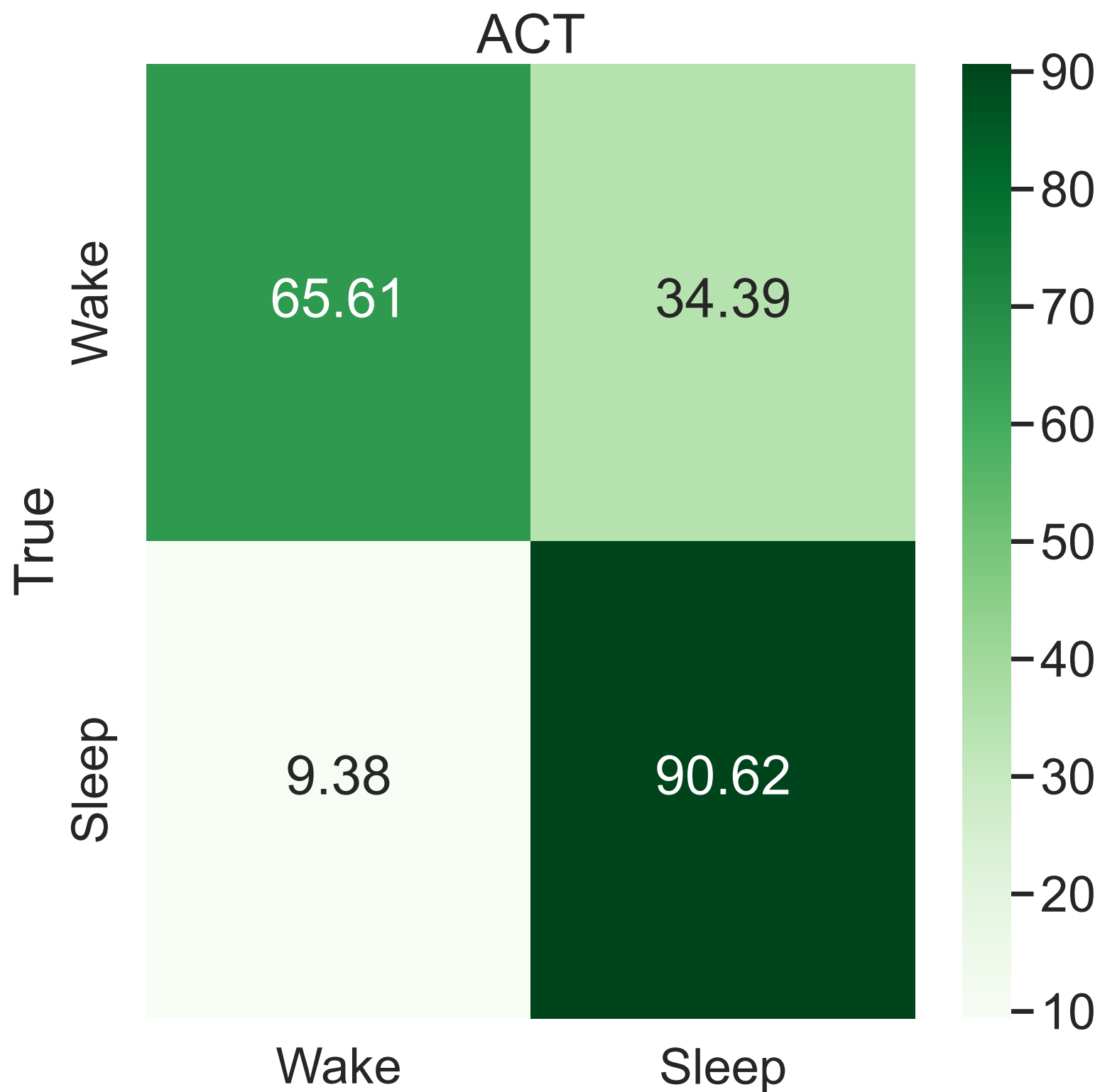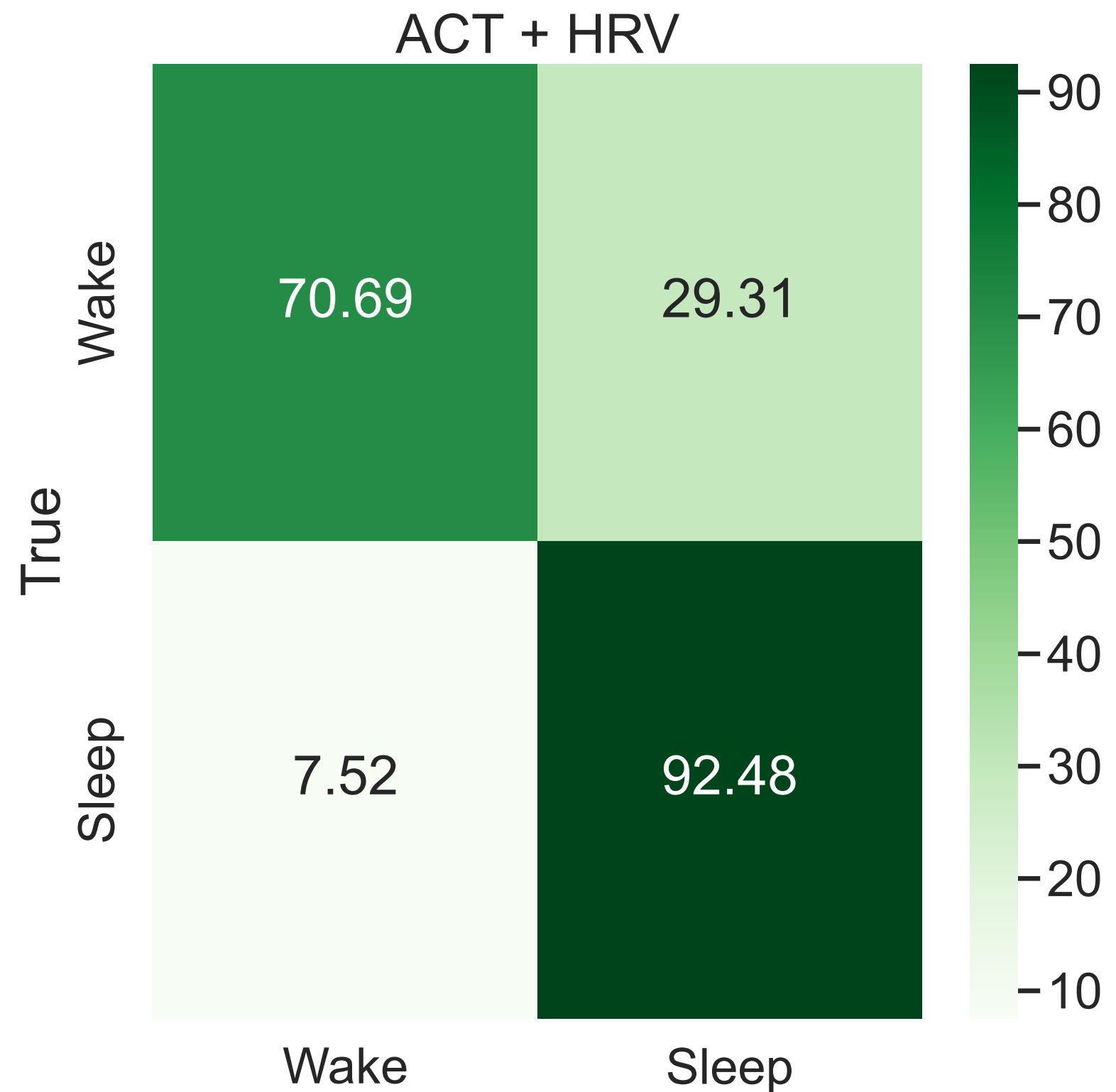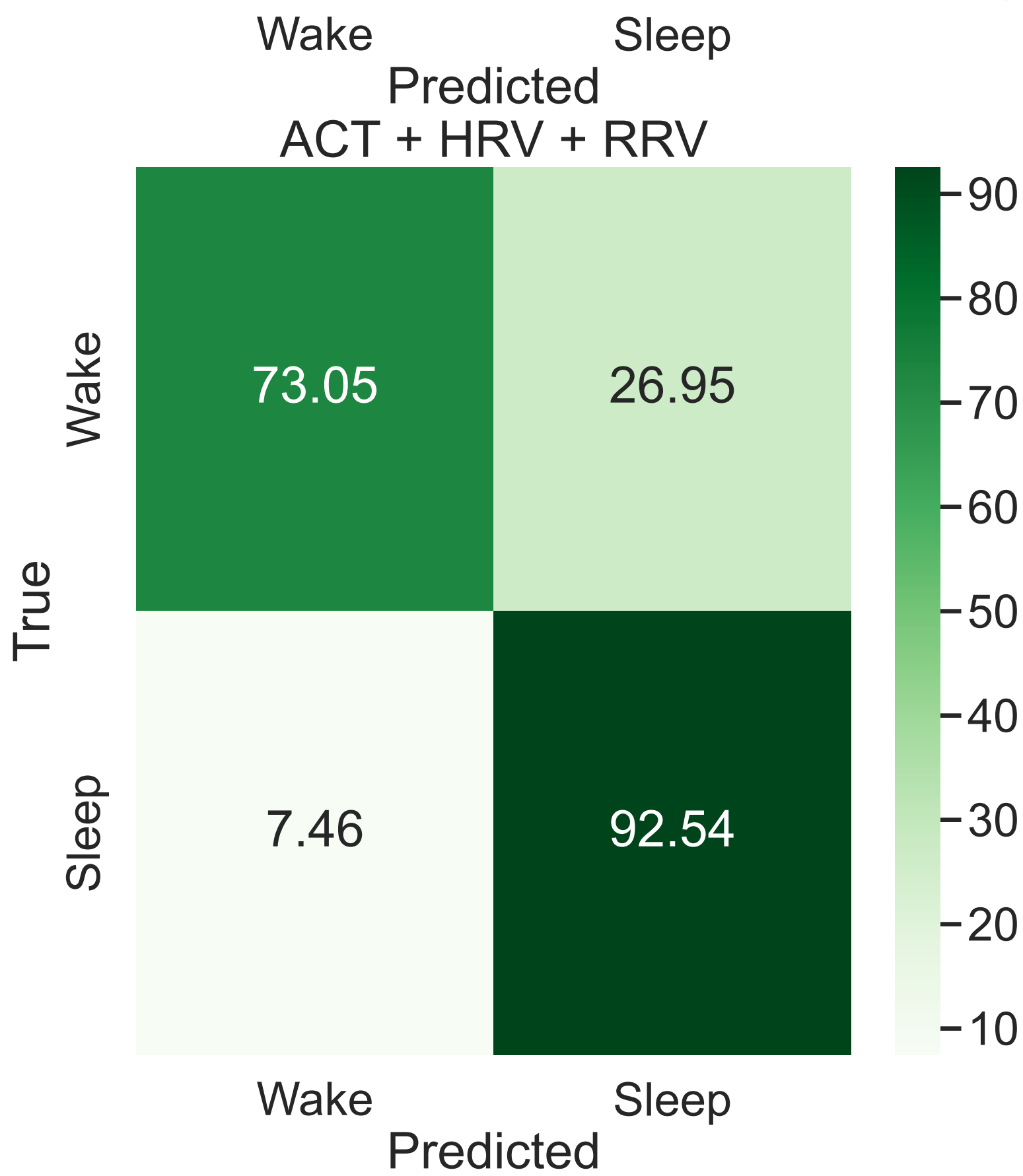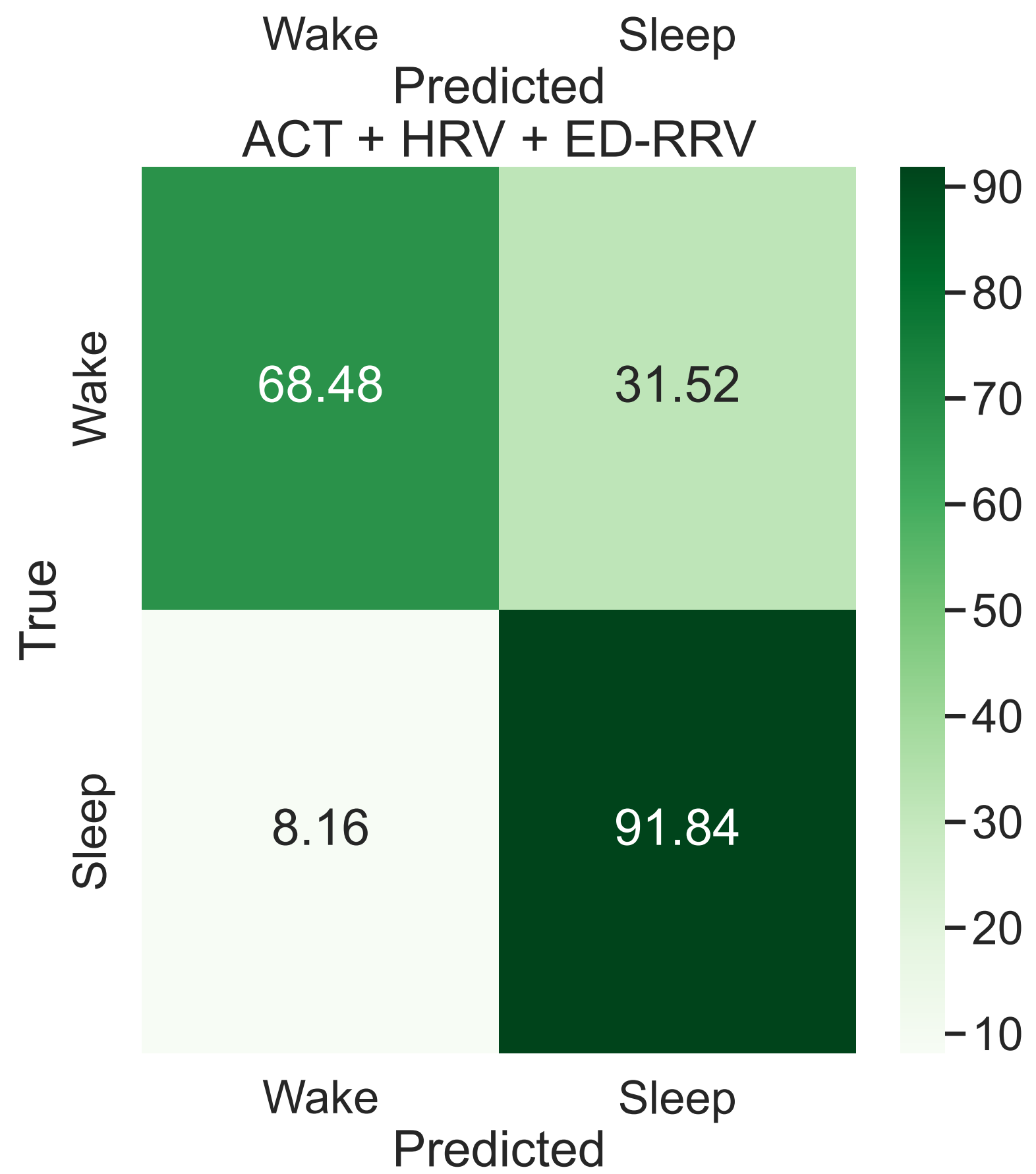

Supplement: zsaf091_suppl_Supplementary_Tables_S1-S8_Figures_S1-S8 [file zsaf091_suppl_supplementary_tables_s1-s8_figures_s1-s8.zip › Sleep_Stage_Classification_large_dataset_supplementary_material/Figure_S5_confusionmatrix_LSTM_binary.pdf]

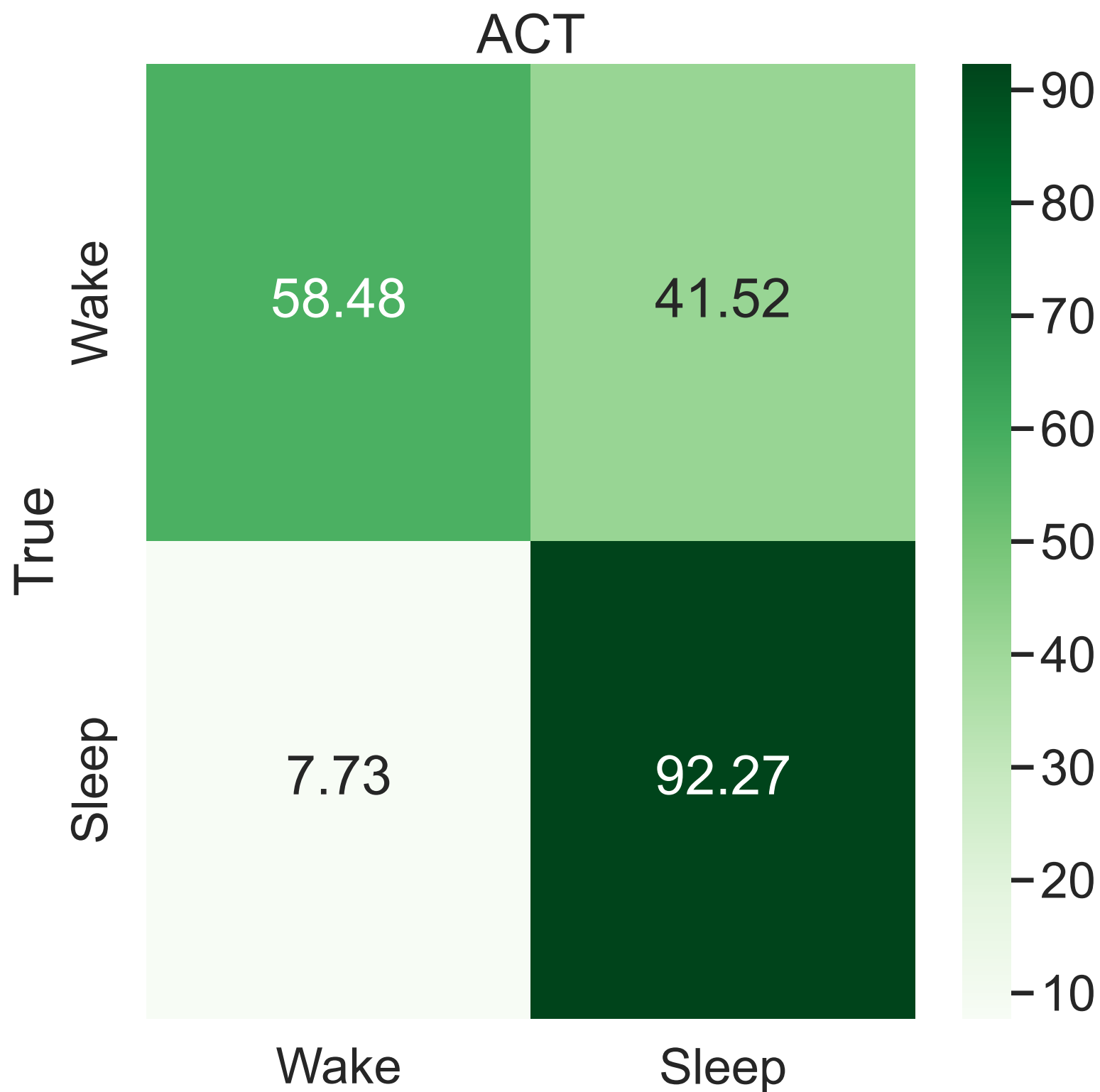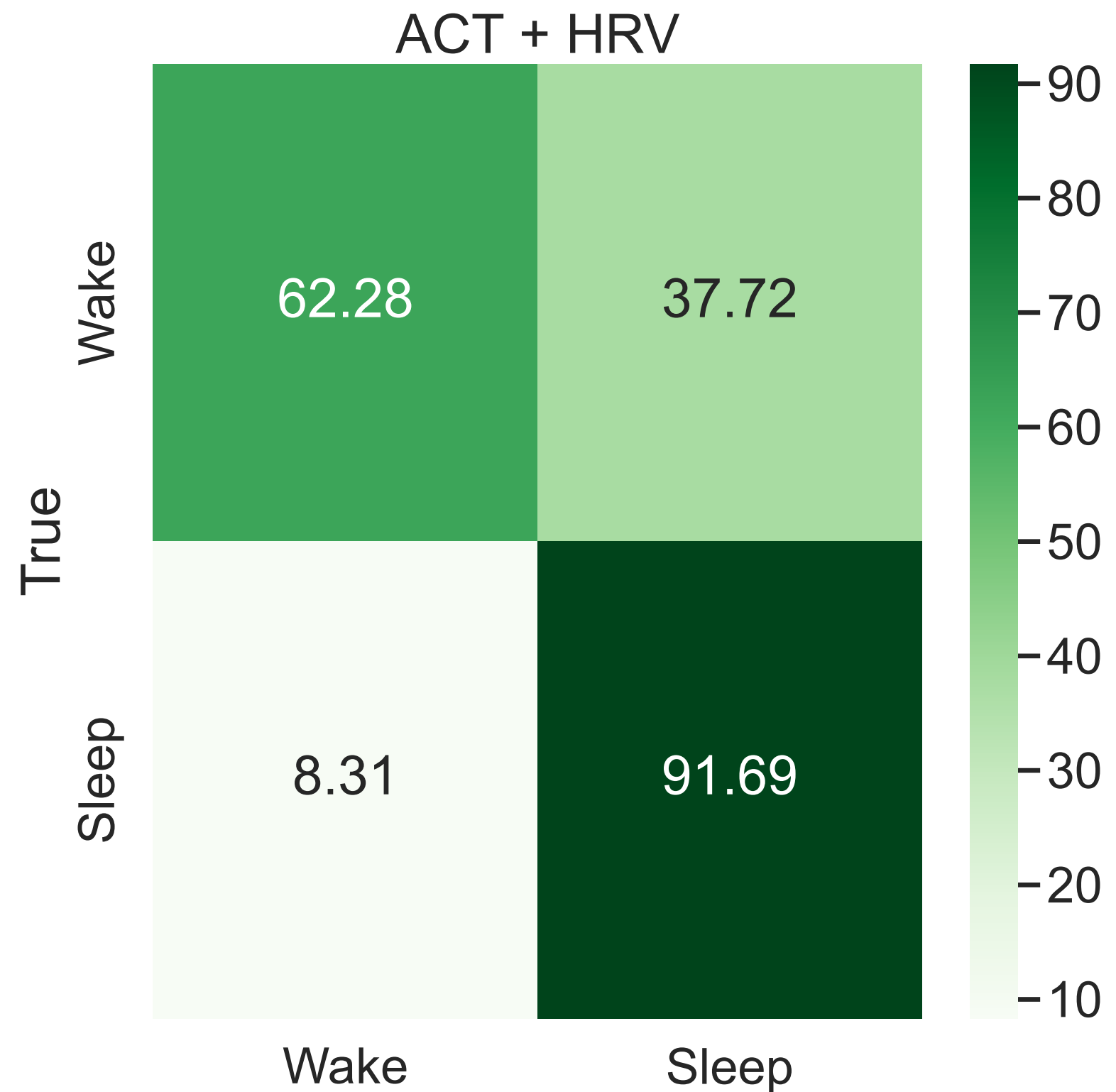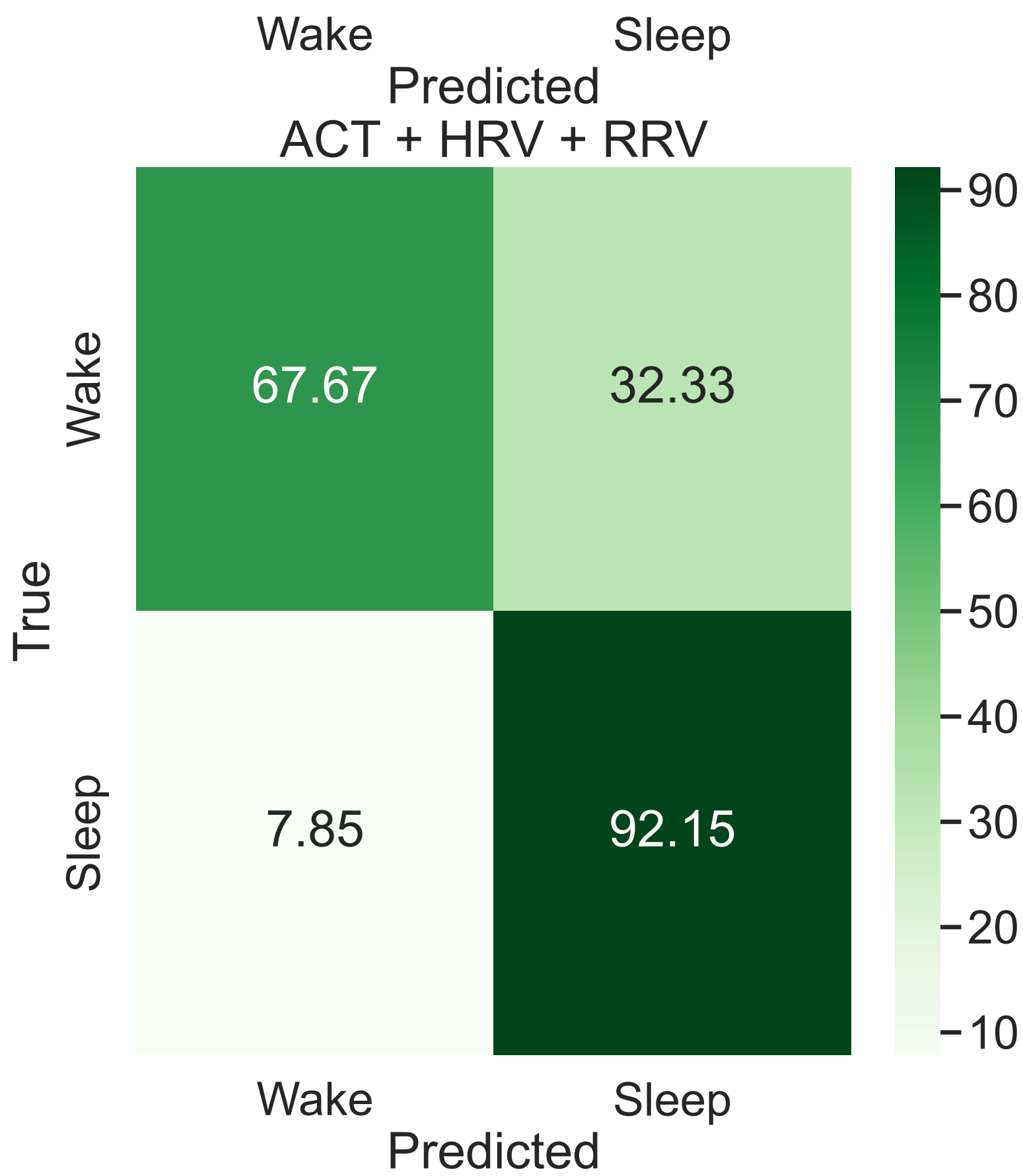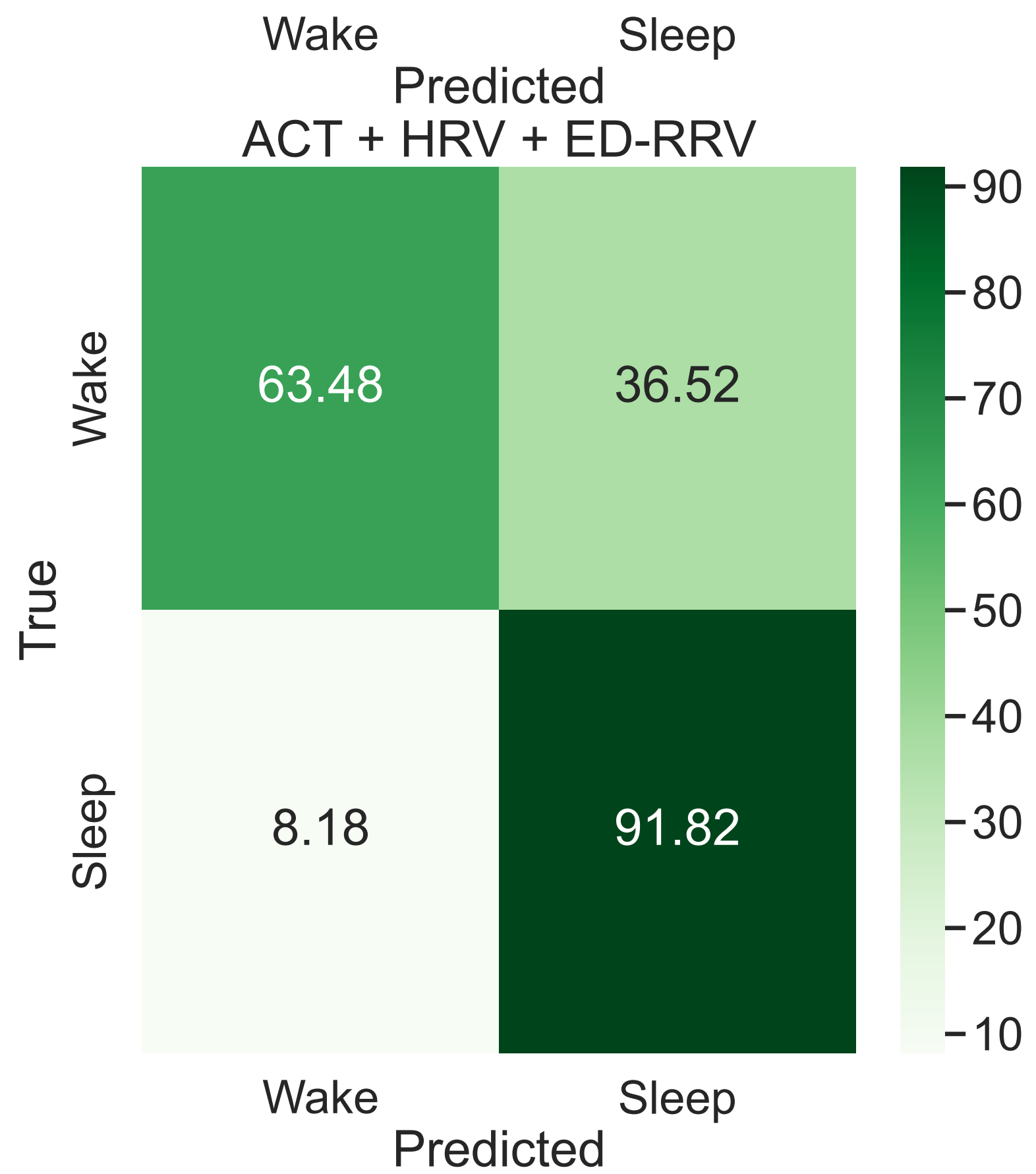

Supplement: zsaf091_suppl_Supplementary_Tables_S1-S8_Figures_S1-S8 [file zsaf091_suppl_supplementary_tables_s1-s8_figures_s1-s8.zip › Sleep_Stage_Classification_large_dataset_supplementary_material/Figure_S6_confusionmatrix_xgb_binary.pdf]

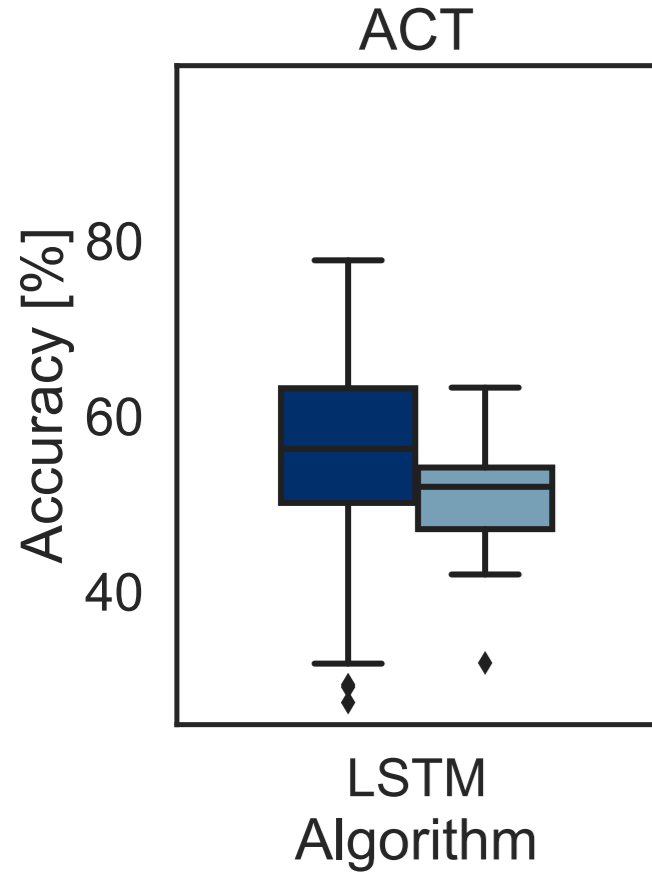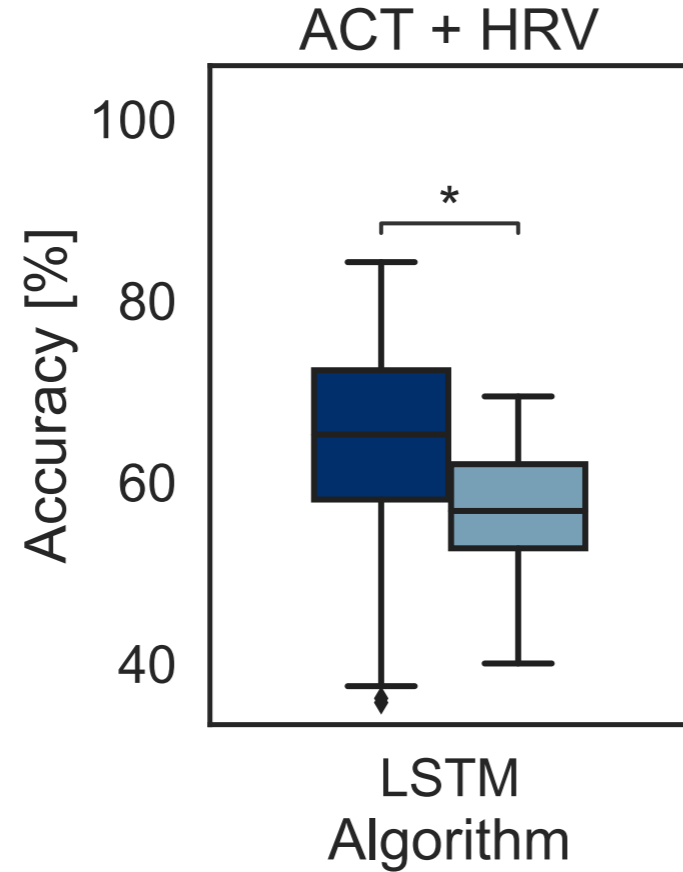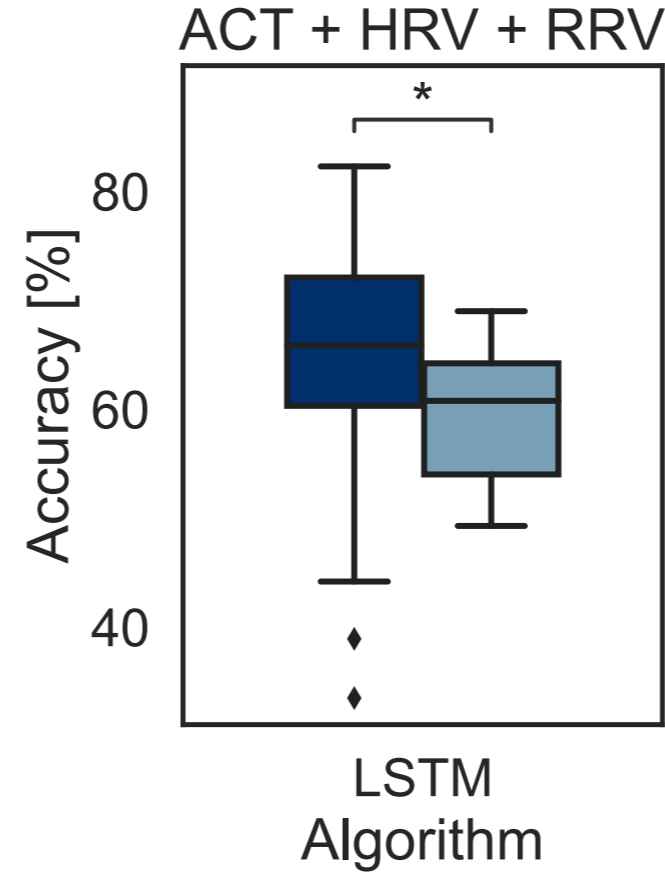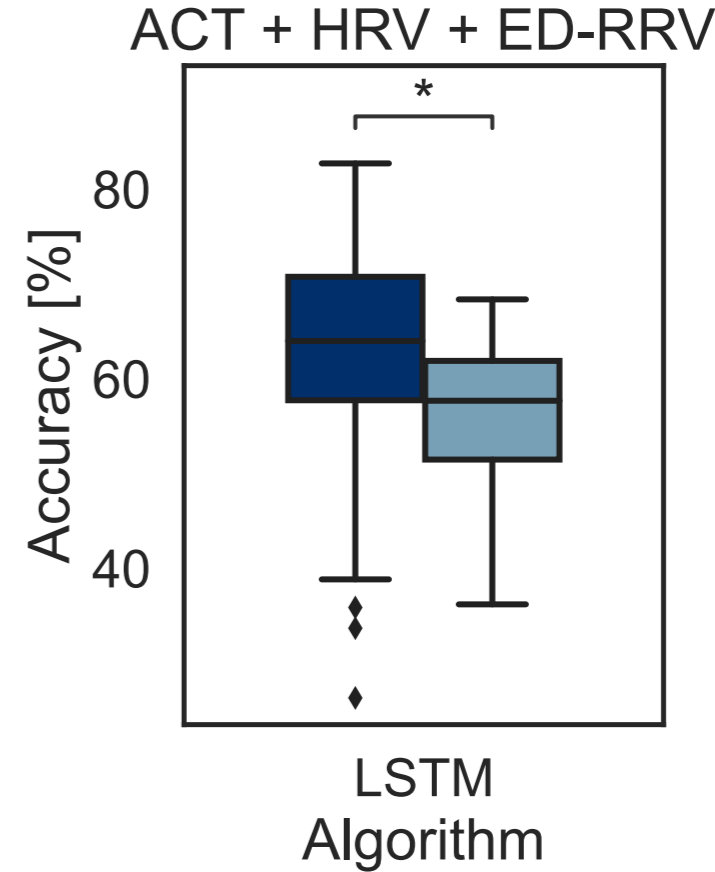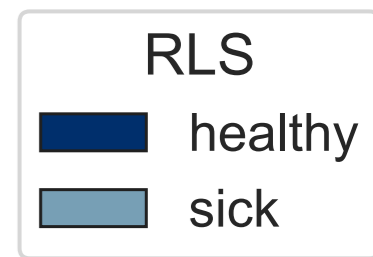

Supplement: zsaf091_suppl_Supplementary_Tables_S1-S8_Figures_S1-S8 [file zsaf091_suppl_supplementary_tables_s1-s8_figures_s1-s8.zip › Sleep_Stage_Classification_large_dataset_supplementary_material/Figure_S7_rls_statistics.pdf]

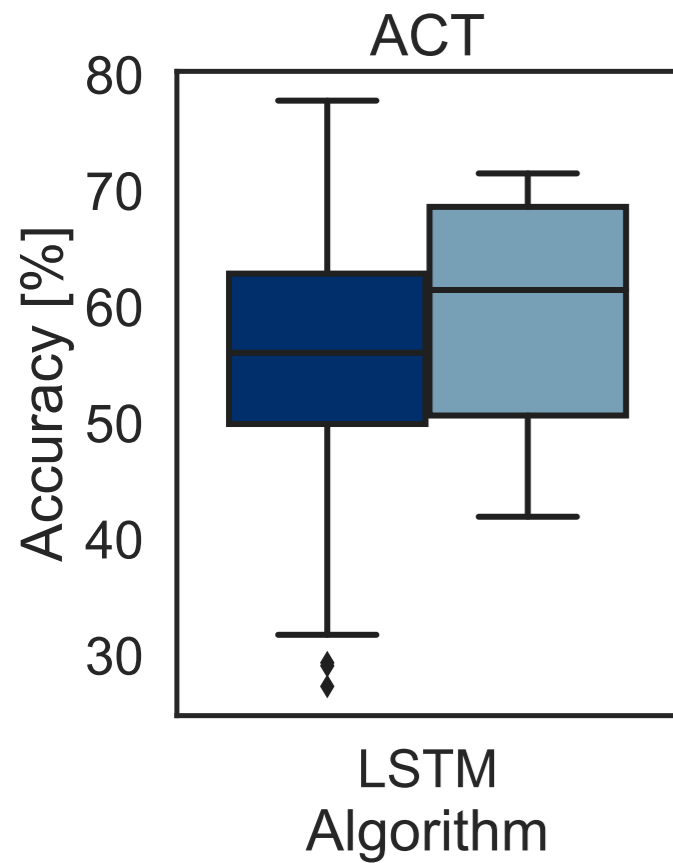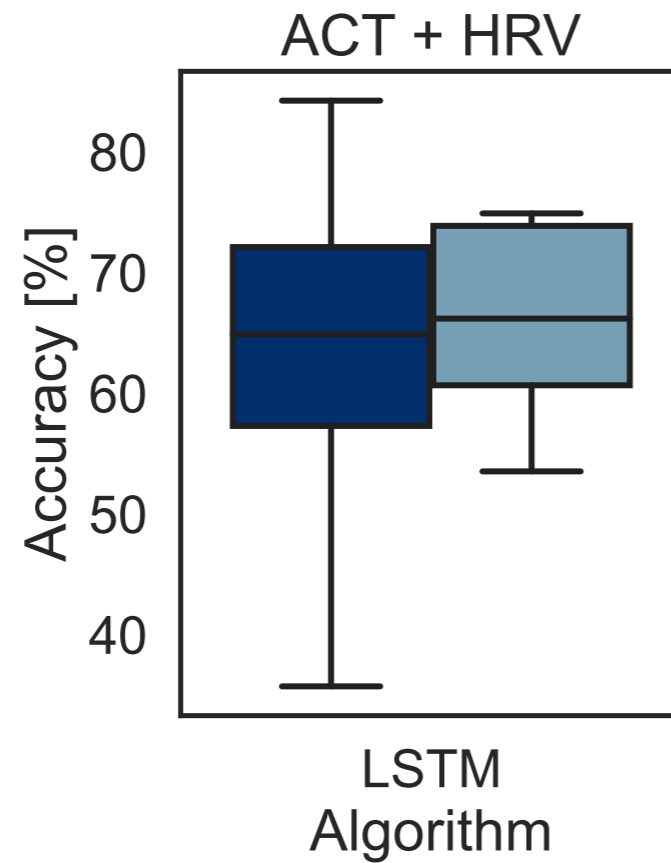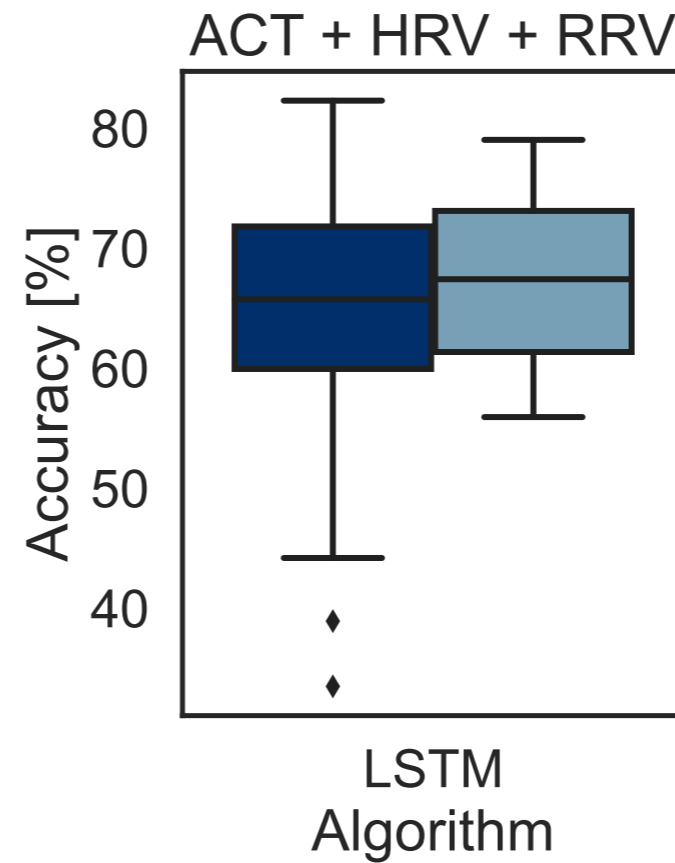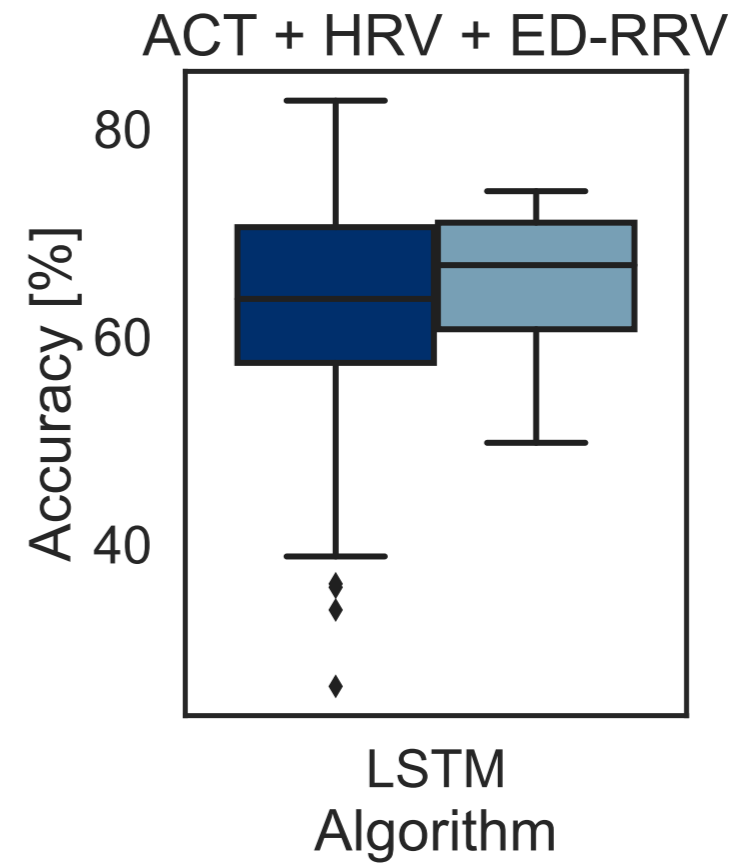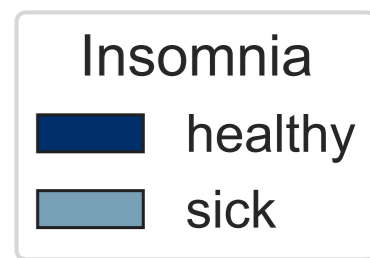

Supplement: zsaf091_suppl_Supplementary_Tables_S1-S8_Figures_S1-S8 [file zsaf091_suppl_supplementary_tables_s1-s8_figures_s1-s8.zip › Sleep_Stage_Classification_large_dataset_supplementary_material/Figure_S8_Insomnia_statistics.pdf]
